# Theoretical foundations of studying criticality in the brain

**DOI:** 10.1162/netn_a_00269

**Published:** 2022-10-01

**Authors:** Yang Tian, Zeren Tan, Hedong Hou, Guoqi Li, Aohua Cheng, Yike Qiu, Kangyu Weng, Chun Chen, Pei Sun

**Affiliations:** Department of Psychology & Tsinghua Laboratory of Brain and Intelligence, Tsinghua University, Beijing, China; Laboratory of Advanced Computing and Storage, Central Research Institute, 2012 Laboratories, Huawei Technologies Co. Ltd., Beijing, China; Institute for Interdisciplinary Information Science, Tsinghua University, Beijing, China; UFR de Mathématiques, Université de Paris, Paris, France; Institute of Automation, Chinese Academy of Science, Beijing, China; University of Chinese Academy of Science, Beijing, China; Tsien Excellence in Engineering Program, School of Aerospace Engineering, Tsinghua University, Beijing, China

**Keywords:** Nonequilibrium criticality, Neural avalanches, Neural dynamics, Directed percolation

## Abstract

Criticality is hypothesized as a physical mechanism underlying efficient transitions between cortical states and remarkable information-processing capacities in the brain. While considerable evidence generally supports this hypothesis, nonnegligible controversies persist regarding the ubiquity of criticality in neural dynamics and its role in information processing. Validity issues frequently arise during identifying potential brain criticality from empirical data. Moreover, the functional benefits implied by brain criticality are frequently misconceived or unduly generalized. These problems stem from the nontriviality and immaturity of the physical theories that analytically derive brain criticality and the statistic techniques that estimate brain criticality from empirical data. To help solve these problems, we present a systematic review and reformulate the foundations of studying brain criticality, that is, ordinary criticality (OC), quasi-criticality (qC), self-organized criticality (SOC), and self-organized quasi-criticality (SOqC), using the terminology of neuroscience. We offer accessible explanations of the physical theories and statistical techniques of brain criticality, providing step-by-step derivations to characterize neural dynamics as a physical system with avalanches. We summarize error-prone details and existing limitations in brain criticality analysis and suggest possible solutions. Moreover, we present a forward-looking perspective on how optimizing the foundations of studying brain criticality can deepen our understanding of various neuroscience questions.

## Author Summary

The brain criticality hypothesis is one of the most focused and controversial topics in neuroscience and biophysics. This research develops a unified framework to reformulate the physics theories of four basic types of brain criticality, ordinary criticality (OC), quasi-criticality (qC), self-organized criticality (SOC), and self-organized quasi-criticality (SOqC), into more accessible and neuroscience-related forms. For the statistic techniques used to validate the brain criticality hypothesis, we also present comprehensive explanations of them, summarize their error-prone details, and suggest possible solutions. This framework may help resolve potential controversies in studying the brain criticality hypothesis, especially those arising from the misconceptions about the theoretical foundations of brain criticality.

## INTRODUCTION

Neuroscience is dawning upon revealing physics foundations of the brain (Abbott, 2008). Ever since the 1970s, the term neurophysics has been suggested as a term to indicate the essential role of physics in understanding the brain (Scott, 1977). More recently, substantial progress has been accomplished in studying brain connectivity and brain functions with statistical physics theories (Lynn & Bassett, 2019).

For brain connectivity, physics provides insights for its emergence, organization, and evolution. Random graphs (Betzel et al., 2016; Betzel & Bassett, 2017a), percolation (Breskin, Soriano, Moses, & Tlusty, 2006; Guo et al., 2021), and other physics theories of correlated systems (Haimovici, Tagliazucchi, Balenzuela, & Chialvo, 2013; Wolf, 2005) are applied to reveal the underlying mechanisms accounting for the origins of brain network properties. Complex network theories act as the foundation of characterizing brain connectivity organizational features (e.g., community; Betzel & Bassett, 2017b; Betzel, Medaglia, & Bassett, 2018; Khambhati, Sizemore, Betzel, & Bassett, 2018), hub (Deco, Tononi, Boly, & Kringelbach, 2015; Gong et al., 2009), and small-world (Bullmore & Sporns, 2012; Deco et al., 2015; structures) and embedding attributes into physical space (Bassett et al., 2010; Kaiser & Hilgetag, 2006). Network evolution driven by neural plasticity helps to explain the dynamics of brain connectivity structures during information processing (Del Pozo et al., 2021; Galván, 2010; Montague, Dayan, & Sejnowski, 1996; Robert & Vignoud, 2021; Song, Miller, & Abbott, 2000). For brain functions, physics presents possible explanations for the origin of information processing capacities from collective neural activities. From single neuron dynamics models (Gerstner, Kistler, Naud, & Paninski, 2014), stochastic network models of neural populations and circuits (Tian, Li, & Sun, 2021; Tian & Sun, 2021), mean-field neural mass models of brain regions (David & Friston, 2003; Touboul, Wendling, Chauvel, & Faugeras, 2011), eventually to models of entire brain networks (Hopfield, 1982; Schneidman, Berry, Segev, & Bialek, 2006), important efforts have been devoted to characterize information-processing-related neural dynamics across different scales. Networks with memory capacities (e.g., Hopfield networks; Tyulmankov, Fang, Vadaparty, & Yang, 2021), which are equivalent to Ising models under specific conditions (Lynn & Bassett, 2019), have been applied to study neural information storage and recall (Haldeman & Beggs, 2005; Krotov & Hopfield, 2020), adaptation to environment changes (Shew et al., 2015), information transmission optimization (Beggs & Plenz, 2003), dynamic range maximization (Kinouchi & Copelli, 2006; Shew, Yang, Petermann, Roy, & Plenz, 2009), and neural computation power (Bertschinger & Natschläger, 2004). These models are further related to maximum entropy models (e.g., specific fine-tuned Ising models) that predict long-range correlations observed among neurons (Ganmor, Segev, & Schneidman, 2011; Schneidman et al., 2006). Moreover, general theories of free-energy principle (Friston, 2009, 2010; Guevara, 2021) and information thermodynamics (Capolupo, Freeman, & Vitiello, 2013; Collell & Fauquet, 2015; Sartori, Granger, Lee, & Horowitz, 2014; Tian & Sun, 2022) are suggested as the unified foundations of perception, action, and learning in the brain.

If one needs to specify one of the most focused and controversial topics among all the works mentioned above, brain criticality may be a potential candidate (Beggs & Timme, 2012). The hypothesis of the critical brain has received increasing attention in recent decades, serving as a possible mechanism underlying various intriguing but elusive phenomena in the brain. In light of our limited understanding of the complex nature of collective neural dynamics, these phenomena include, to name a few, efficient transitions between cortical states (Fontenele et al., 2019), maximal dynamic ranges of neural responses (Antonopoulos, 2016; Gautam, Hoang, McClanahan, Grady, & Shew, 2015; Kinouchi & Copelli, 2006; Shew et al., 2009), optimized information transmission and representation (Antonopoulos, 2016; X. Li & Small, 2012; Shew, Yang, Yu, Roy, & Plenz, 2011), and numerous other issues concerning brain functions that we have mentioned above. One can see Beggs (2007), Chialvo (2010), Cocchi, Gollo, Zalesky, and Breakspear (2017), Hesse and Gross (2014), and Shew and Plenz (2013) for systematic reviews of the diverse function advantages implied by brain criticality and their experimental demonstrations. From a Darwinian perspective, one potential reason for the brain to feature criticality lay in that the most informative parts of external world principally occur at a borderline between purely ordered and purely disordered states (information would be trivial in a purely ordered world while it would be incomprehensible in a purely disordered world). Becoming critical may be a potential way for the brain to adapt to the complex world, where nontrivial information has a finite opportunity to occur (Bak, 2013; Chialvo, 2010). To date, generic features of a critical brain with the characteristics discussed above, such as divergent correlation length, neuronal avalanches with power law behaviors, and long-range correlations on the microscopic scale (e.g., neural populations), have been extensively observed in mathematical models in conjunction with experimental data (e.g., Beggs & Plenz, 2003; Dalla Porta & Copelli, 2019; Fosque, Williams-García, Beggs, & Ortiz, 2021; Gireesh & Plenz, 2008; Hardstone, Mansvelder, & Linkenkaer-Hansen, 2014; Petermann et al., 2009; Poil, Hardstone, Mansvelder, & Linkenkaer-Hansen, 2012; Poil, van Ooyen, & Linkenkaer-Hansen, 2008; Ponce-Alvarez, Jouary, Privat, Deco, & Sumbre, 2018; G. Scott et al., 2014; Shew et al., 2009; Shriki et al., 2013; Tagliazucchi, Balenzuela, Fraiman, & Chialvo, 2012; Tkačik et al., 2015).

Our work does not aim at repeatedly reviewing experimental advances concerning brain criticality and its biological significance, given that they have been comprehensively summarized by existing reviews (Beggs, 2007; Chialvo, 2010; Cocchi et al., 2017; Hesse & Gross, 2014; Muñoz, 2018; Shew & Plenz, 2013). On the contrary, our motivation is to present a systematic and accessible review of the theoretical methods applied to achieve these advances, which have not received necessary attention yet.

These theoretical foundations are initially thought to be incomprehensible and irrelevant to neuroscience. However, practice suggests that omitting these physical and mathematical backgrounds does not significantly improve the accessibility of studies on brain criticality. Instead, the lack of detailed explanations of theoretical foundations has frequently misled neuroscientists, leading to diverse confusions about the precise meaning, identification criteria, and biological corollaries of brain criticality. As a result, criticality, an analytic statistical physics theory with solid foundations, unnecessarily becomes an elusive black box for neuroscientists. To address this issue, we use the terminology of neuroscience to present a self-contained framework of brain criticality, reviewing and reformulating (1) physical theories that analytically derive brain criticality and (2) statistic techniques that computationally estimate brain criticality from empirical data. Given the frequent misunderstanding of neural avalanches, our discussions primarily focus on brain criticality analysis on the microscopic scale of the brain. The objectives guiding our review are tripartite: (1) explaining why brain criticality matters in the brain, (2) understanding what is brain criticality and what it conveys about the brain, and (3) confirming how to identify potential brain criticality and ensure the validity of analyses.

## BRAIN CRITICALITY: GENERAL CONCEPTS

### Overview of Brain Criticality

Brain criticality frequently confuses neuroscientists since too many distinct phenomena are studied under this name without being properly classified. In this review, brain criticality refers to a family of critical processes in neural dynamics where erratic fluctuations appear to reduce dynamic stability. To present a systematic classification framework, we discuss three fundamental perspectives concerning brain criticality. Table 1 provides all the necessary glossaries in comprehensible forms.

**Table 1.** Key concepts in describing brain criticality

Concept	Meaning
Equilibrium	A case where the system maximizes entropy and conserves energy simultaneously. The stationary probability distribution 𝒫_*eq*_(·) of system states of a system at equilibrium is the Boltzmann distribution. At equilibrium, the transition dynamics between system states *c* and *c*′ satisfies the detailed balance condition 𝒫_*eq*_(*c*) 𝒲(*c* → *c*′) = 𝒫_*eq*_(*c*′) 𝒲(*c*′ → *c*), where 𝒲(· → ·) denotes the transition probability.
Nonequilibrium	A case where the system is out of equilibrium because the transition dynamics between system states breaks the detailed balance condition. In other words, the transition dynamics between states becomes directional rather than symmetric.
Self-organization	A process where the internal complexity of a system increases without being tuned by any external mechanism. All potentially emergent properties are created by endogenous feedback processes or other internal factors inside the system.
Criticality	A kind of phenomena where the systems is generally close to specific critical points separating between multiple system states. Small disturbances are sufficient to make the system experience dramatic and sharp transitions between system states.
Quasi-criticality	A kind of phenomena where all statistical physics relations required by criticality are principally adhered by the system but slight and inconstant deviations from perfect criticality can be seen on the actual values of characteristic variables. These deviations robustly exist and are generally independent of data noises.
Sub-criticality	A kind of system states below criticality. They occur when the order parameter (i.e., the macroscopic observable used to describe system states) remains at zero even with the addition of derives, corresponding to disordered system dynamics.
Super-criticality	A kind of system states above criticality. They occur when the order parameter is positive, corresponding to ordered system dynamics.

#### Being nonequilibrium.

**Figure 1.**
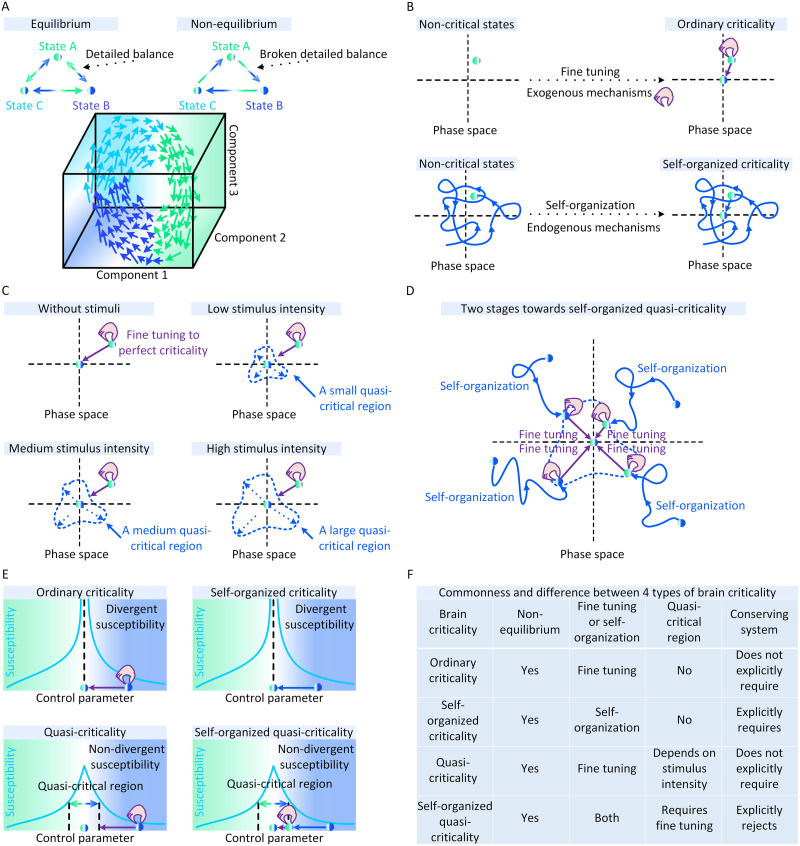
Conceptual illustrations of brain criticality. (A) Difference between equilibrium and nonequilibrium dynamics in a three-state brain (upper parallel). Brain states are characterized by three system components. We illustrate an instance of nonequilibrium dynamics between these states (bottom parallel). (B) Fine tuning with exogenous mechanisms (represented by animated hands) makes the brain evolve from a noncritical state (upper left) to the critical state (upper right). Endogenous mechanisms enable the brain to self-organize from a noncritical state (bottom left) to the critical state (bottom right). (C) Increasing stimulus intensity enlarges the quasi-critical region around the perfect critical point in a quasi-critical system. (D) The approaching process to a critical point in a self-organized quasi-critical system consists of two stages. In the first stage, the brain self-organizes from a noncritical state to a quasi-critical region based on certain endogenous mechanisms. In the second stage, additional exogenous mechanisms are necessary to fine tune the brain to the critical point. Otherwise, the brain just hovers within the quasi-critical region. (E) The difference between four types of brain criticality from the perspective of susceptibility. For standard brain criticality (e.g., ordinary criticality and self-organized criticality), susceptibility becomes divergent (i.e., infinite) at the critical point. For nonstandard brain criticality (e.g., quasi-criticality and self-organized quasi-criticality), susceptibility is always nondivergent (i.e., finite). The quasi-critical region is defined as a set of all control parameters where susceptibility values are no less than a specific threshold (e.g., half-maximum value). (F) The commonness and difference between four types of brain criticality.

First, the brain, similar to other biological systems, generally exhibits temporal evolution from initial states that are far away from equilibrium (Gnesotto, Mura, Gladrow, & Broedersz, 2018; Lynn, Cornblath, Papadopoulos, Bertolero, & Bassett, 2021). These departures from equilibrium arise due to diverse endogenous causes (Gnesotto et al., 2018; Perl et al., 2021) to break the detailed balance to support consciousness, sensing, and adaptation (Lynn et al., 2021; Perl et al., 2021). Therefore, potential critical phenomena underlying neural dynamics, at least in most neural dynamics models and empirical datasets, are basically nonequilibrium and cannot be characterized by equilibrium statistic mechanics. In Figure 1A, we illustrate the difference between equilibrium and nonequilibrium dynamics.

#### Fine tuning versus self-organization.

Second, there exist two types of general mechanisms underlying the existence of brain criticality. One type of mechanisms either arise from the external manipulations outside the brain (e.g., researchers manipulate the tonic dopamine D1-receptor stimulation; Stewart & Plenz, 2006, 2008) or adjust network topology (Kaiser & Hilgetag, 2010; Rubinov, Sporns, Thivierge, & Breakspear, 2011; S. Wang & Zhou, 2012) or belong to the top-down biological processes that globally function on neural dynamics inside the brain (e.g., anesthesia effects; Fontenele et al., 2019; Hahn et al., 2017; Ribeiro et al., 2010) as well as sleep restoration effects (Meisel, Olbrich, Shriki, & Achermann, 2013). Neural dynamics is passively fine-tuned toward or away from ordinary criticality (OC) by these exogenous mechanisms, similar to ordinary critical phenomena that require the fine tuning of order parameters.

Another type of mechanisms includes all endogenous factors of neural dynamics (e.g., neural plasticity mechanisms such as spike-timing dependent synaptic plasticity (Effenberger, Jost, & Levina, 2015; Meisel & Gross, 2009; Shin & Kim, 2006), short-term synaptic plasticity (Levina, Herrmann, & Geisel, 2007, 2009), retro-synaptic signals (Hernandez-Urbina & Herrmann, 2017), and Hebbian rules (De Arcangelis & Herrmann, 2010; De Arcangelis, Perrone-Capano, & Herrmann, 2006), which locally function on neural dynamics as drive and dissipation components. The interactions between these components naturally form feedback control loops to support the self-organization of neural dynamics toward the critical point (Beggs, 2007; Chialvo, 2010). This spontaneously emerged brain criticality, distinct from ordinary critical phenomena, is conjectured as a kind of self-organized criticality (SOC) (Chialvo, 2010). In Figure 1B, we present conceptual illustrations of ordinary criticality and self-organized criticality in the brain.

#### Standard versus nonstandard.

Third, brain criticality frequently occurs in nonstandard forms due to stimulus drives or endogenous factors. On the one hand, slight and inconstant deviations from perfect brain criticality can be seen on the actual values of characteristic variables, differentiating the characterized phenomena from the standard criticality (Fosque et al., 2021; Williams-García, Moore, Beggs, & Ortiz, 2014). On the other hand, all statistical physics relations required by perfect brain criticality are still adhered by these actual characteristic variables, distinguishing the brain from being noncritical (Fosque et al., 2021; Williams-García et al., 2014).

**Figure 2.**
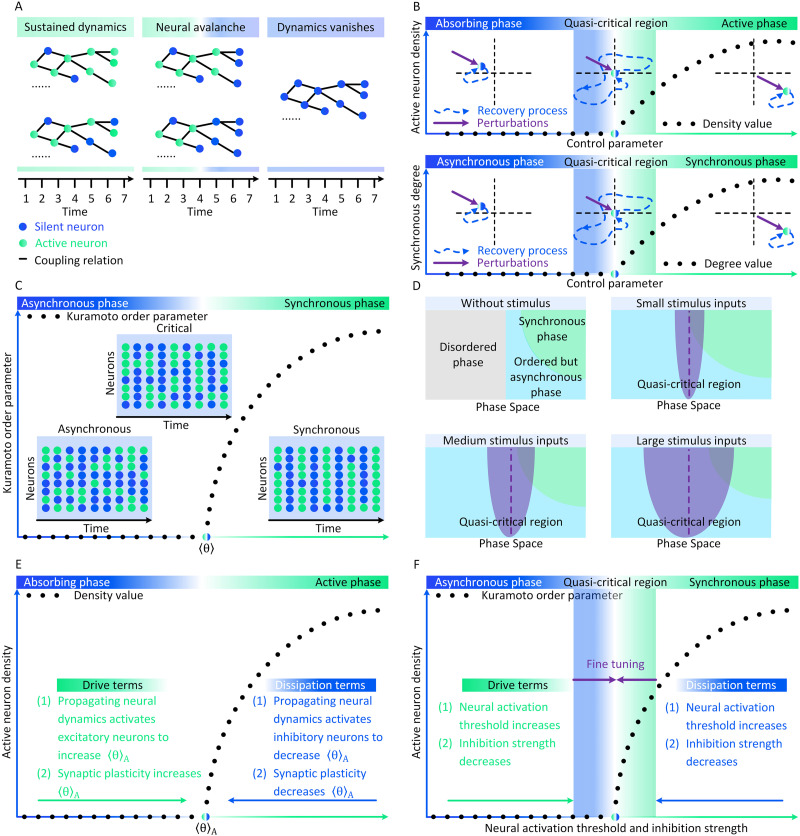
Conceptual illustrations of the relations between neural avalanches and brain criticality. (A) Instances of neural avalanche, self-sustained neural dynamics, and vanished neural dynamics. (B) The recovery processes of brain states after the same perturbation in the space of absorbing and active phases (upper parallel) and the space of synchronous and asynchronous phases (bottom parallel). The recovery processes after perturbations are relatively fast when the brain is far from the critical point or the quasi-critical region, These recovery processes slow down when the brain is close to the critical point or the quasi-critical region. (C) The conceptual illustrations of neural dynamics when the brain state is asynchronous, synchronous, or at ordinary criticality. (D) Without stimuli, there initially exist disordered (gray), ordered but asynchronous (light blue), synchronous (green) phases in the phase space of the brain. Stimulus inputs imply quasi-criticality in the brain. An increasing stimulus intensity enlarges the quasi-critical region (purple) around the Widom line (purple dashed line). (E) The conceptual illustrations of how endogenous mechanisms in conserved neural dynamics can function as drive or dissipation terms to create self-organized criticality between absorbing and active phases in the brain. (F) In the self-organized quasi-critical brain, endogenous mechanisms in nonconserved neural dynamics only support the self-organization towards a quasi-critical region between asynchronous and synchronous phases. Extra exogenous mechanisms are required to fine tune the brain towards the critical point.

For ordinary criticality, its nonstandard form is referred to as quasi-criticality (qC) (Fosque et al., 2021; Williams-García et al., 2014). Diverse mechanisms can force the brain to depart from perfect ordinary criticality and exhibit quasi-critical neural dynamics, among which, stimulus derive may be the most common one (Fosque et al., 2021; Williams-García et al., 2014). In general, sufficiently strong stimulus drives can capture or even govern neural dynamics. Similar to the situation where external inputs suppress irregular neural dynamics (Molgedey, Schuchhardt, & Schuster, 1992), the stimuli that are too strong may evoke intense but less changeable neural dynamics to make the brain depart from the perfect critical point (Fosque et al., 2021; Williams-García et al., 2014). Let us take the qC phenomenon introduced by Fosque et al. (2021) and Williams-García et al. (2014) as an instance. Under specific conditions, the actual brain state may be close to a Widom line in the three-dimensional space defined by the stimulus intensity *υ*, refractory period length *τ*, and branching ratio *κ* (i.e., the time-dependent average number of subsequent neural activities caused by a single neuron activation event (Haldeman & Beggs, 2005). The Widom line is a line of all the combinations of (*υ*, *τ*, *κ*) where the susceptibility of neural dynamics is maximized (Fosque et al., 2021; Williams-García et al., 2014). The susceptibility is defined by lim_*x*→0_ $\frac{\partial y}{\partial x}$, where *y* is the neural dynamics state and *x* denotes a factor that affects *y*. In general, one can understand susceptibility as the degree to which fluctuations in the state of each neuron can propagate to neighbored neurons (Williams-García et al., 2014). Being close to the Widom line suggests the existence of quasi-criticality in the brain. Moving along the Widom line as the stimulus intensity increases, the susceptibility of neural dynamics decreases, and the branching ratio at maximal susceptibility will decrease as well (Fosque et al., 2021; Williams-García et al., 2014). Significant deviations from the Widom line suggest noncriticality (i.e., the subcriticality where neural dynamics is disordered and the super-criticality where neural dynamics is ordered; Williams-García et al., 2014). In Figure 1C, we conceptually illustrate how stimuli imply qC in the brain. In Figure 2D, the qC phenomenon in Fosque et al. (2021) and Williams-García et al. (2014) is shown in detail.

As for self-organized criticality (SOC), its nonstandard form is defined according to statistical physics criteria. Perfect self-organized criticality only exists in conserved neural dynamics (e.g., see integrate-and-fire neurons analyzed by Levina et al., 2007), where system energy (i.e., neural activities) either conserves within the system and only dissipates at the system boundary, or dissipates inside the system (i.e., bulk dissipation) with a dissipation rate vanishing in the system size limit (Malcai, Shilo, & Biham, 2006). Under more general conditions where neural dynamics are not conserved (e.g., see leaky integrate-and-fire neurons analyzed by Levina et al., 2007; Millman, Mihalas, Kirkwood, & Niebur, 2010; Rubinov et al., 2011; Stepp, Plenz, & Srinivasa, 2015; where neural dynamics dissipates within the system due to voltage leak), perfect self-organized criticality can be broken by any rate of bulk dissipation (Bonachela, De Franciscis, Torres, & Muñoz, 2010; Bonachela & Muñoz, 2009; Buendía, di Santo, Villegas, Burioni, & Muñoz, 2020b; de Andrade Costa, Copelli, & Kinouchi, 2015). Stronger bulk dissipation implies larger deviations from perfect self-organized criticality (De Arcangelis et al., 2006). Consequently, the self-organization process of nonconserved neural dynamics only make the brain hover around the critical point. Any further closeness toward the critical point requires the fine tuning of order parameter by additional exogenous mechanisms, which is different from pure self-organized criticality (Bonachela et al., 2010; Bonachela & Muñoz, 2009; Buendía et al., 2020b; de Andrade Costa et al., 2015). This nonconserved self-organization process is termed as self-organized quasi-criticality (SOqC) (Bonachela & Muñoz, 2009). Similar to SOC in conserved dynamics, neural plasticity mechanisms, such as spike-timing-dependent synaptic plasticity (Rubinov et al., 2011), Hebbian rules (De Arcangelis et al., 2006), short-term synaptic depression in conjunction with spike-dependent threshold increase (Girardi-Schappo et al., 2021), and inhibitory plasticity in conjunction with network topology (Ma, Turrigiano, Wessel, & Hengen, 2019), can serve as underlying self-organization mechanisms of SOqC. Because purely conserved neural dynamics is relatively rare in empirical data (e.g., neural dynamics is conserved for integrate-and-fire neurons; Levina et al., 2007; and leaky integrate-and-fire neurons whose presynaptic inputs are exactly equal to the sum of voltage leak and potential costs during neural spiking (Bonachela et al., 2010), we suggest that SOqC may be more common in the brain than SOC. In Figure 1D, we present conceptual instances of the two-stage approaching process towards the critical point in the brain with SOqC.

#### Classification of brain criticality.

The above discussion has presented a classification framework of brain criticality, that is, ordinary criticality (OC), quasi-criticality (qC), self-organized criticality (SOC), and self-organized quasi-criticality (SOqC). In Figure 1E, we compare between these four types of brain criticality in term of susceptibility. In general, susceptibility diverges at the critical point in a brain with standard criticality (e.g., OC and SOC) while it does not diverge in the quasi-critical region of a brain with nonstandard criticality (e.g., qC and SOqC). In Figure 1F, we summarize the commonness and difference between these four types of brain criticality discussed in our review. From a neuroscience perspective, a brain with critical neural dynamics is expected to be near the critical point and prepared for tremendous changes in cortical states during a short duration. This intriguing property coincides with the experimentally observed efficient transitions between cortical states (e.g., Cardin, 2019; Holcman & Tsodyks, 2006; Jercog et al., 2017; H. Lee, Wang, & Hudetz, 2020; Reimer et al., 2014) and, therefore, interests researchers for the potential existence of brain criticality. The importance of identifying brain criticality in neural dynamics is beyond brain criticality itself because it implies an opportunity to explain and predict brain function characteristics by various statistical physics theories built on nonequilibrium criticality.

### Neural Avalanches and Their Phases

To identify potential nonequilibrium criticality in the brain, researchers actually characterize neural dynamics as a physical system with absorbing states and avalanche behaviors (Hinrichsen, 2000; Larremore, Carpenter, Ott, & Restrepo, 2012; Lübeck, 2004). In general, one needs to consider the propagation of neural dynamics where neurons are either activated (“on” state) or silent (”off” state) (Dalla Porta & Copelli, 2019). A silent neuron may be activated with a probability defined by the number of activated presynaptic neurons and the coupling strength *θ* among neurons (e.g., neural correlation; Franke et al., 2016). An activated neuron spontaneously becomes silent at a constant rate (e.g., after the refractory period; Kinouchi & Copelli, 2006; Squire et al., 2012). These definitions naturally support to distinguish between different phases of neural dynamics. Here we review two kinds of phase partition that are active in neuroscience.

#### Absorbing versus active.

The first group of phases are absorbing and active phases (Larremore et al., 2012). The absorbing phase refers to cases where couplings between neurons are weak and all neurons eventually become silent (neural dynamics vanishes). Once a neural dynamics process vanishes, it cannot reappear by itself. The brain requires new drives (e.g., neurons activated spontaneously or by stimuli) to trigger new neural dynamics. The active phase, on the other hand, correspond to cases where the “on” state propagates among neurons with strong couplings, leading to stable self-sustained neural dynamics (e.g., nonzero time- and ensemble-averaged density of active neurons in the brain). In Figure 2A, we show conceptual instances of neural avalanches, self-sustained neural dynamics, and vanished neural dynamics. Denoting *ρ*(*t*) as the density of active neurons at moment *t*, we can simply represent the absorbing (Equation 1) and active (Equation 2) phases of a neural dynamics process triggered by an active neuron at moment 0 as

$$\rho \left(t\right)=0,\;\exists t>0,$$ (1)

$$\rho \left(t\right)>0,\;\forall t>0.$$ (2)

#### Synchronous versus asynchronous.

The second group of phases are synchronous and asynchronous phases (di Santo, Villegas, Burioni, & Muñoz, 2018; Fontenele et al., 2019; Girardi-Schappo et al., 2021). As their names suggest, these two phases correspond to the situations where synchronization emerges or disappears in neural activities, respectively. Synchronization refers to the cases where “on” states appear in an oscillatory, although not strictly periodic, manner. To quantify its potential existence, we can measure the variability of neural dynamics using the coefficient of variation (CV) (di Santo et al., 2018; Fontenele et al., 2019; Girardi-Schappo et al., 2021) or the Kuramoto order parameter (Acebrón, Bonilla, Pérez Vicente, Ritort, & Spigler, 2005; Arenas, Díaz-Guilera, Kurths, Moreno, & Zhou, 2008). CV can be defined from diverse perspectives, yet the most common definition is the ratio between the standard deviation and the mean of the interspike interval length (di Santo et al., 2018; Fontenele et al., 2019; Girardi-Schappo et al., 2021). A higher value of CV implies the reduction of synchronization. For most neural dynamics data, an empirical choice of the CV threshold that separates between synchronous and asynchronous phases may be ≃1 (Fontenele et al., 2019) or ≃$\frac{3}{2}$ (Fontenele et al., 2019). The Kuramoto order parameter *ω* ∈ [0, 1] measures the coherent degree of neural dynamics based on the Kuramoto model of oscillators (for detailed definitions see Acebrón et al., 2005; Arenas et al., 2008). Perfect synchronization emerges when *ω* = 1 and vanishes when *ω* = 0 (Acebrón et al., 2005; Arenas et al., 2008).

#### Critical point or quasi-critical region.

The boundary between these two phases is the critical point, at which the brain is on the edge of exhibiting self-sustained (for absorbing and active phases) or synchronous (for synchronous and asynchronous phases) neural dynamics. Perturbations (e.g., the propagation of “on” state among neurons) to the absorbing or asynchronous phase do not have characteristic lifetime and size. These perturbations, referred to as neural avalanches, are expected to exhibit power law properties in their lifetime (time difference between the first and last activation of neurons in between complete quiescent epochs) and size (number of active neurons along with the excursion) distributions (Hesse & Gross, 2014; Hinrichsen, 2000; Larremore et al., 2012; Lübeck, 2004). In general, the emergence of neural avalanches implies the slowing down of neural dynamics, that is, the brain state recovery process toward the baseline state after fluctuations changes from fast (exponential) to slow (power law) (Cocchi et al., 2017; Hesse & Gross, 2014). The dynamic stability of neural dynamics is limited by the slow recovery and, therefore, cannot robustly counteract perturbations. Consequently, small perturbations initiated on the microscopic scale may still make the brain change sharply on the macroscopic scale (Cocchi et al., 2017; Hesse & Gross, 2014). In Figure 2B, we conceptually illustrate how the recovery process slows down when the brain is close to the critical point or the quasi-critical region.

### General Relations Between Neural Avalanches and Brain Criticality

The relation between neural avalanches and brain criticality is frequently neglected or misunderstood. Neural avalanche data alone is not sufficient to determine the concrete type of brain criticality (i.e., OC, qC, SOC, and SOqC) unless additional information about the mechanisms underlying neural avalanche emergence is provided (e.g., if neural dynamics is conserved or self-organizing). To explore a concrete type of brain criticality, researchers need to explicitly present its definition depending on different control parameters (e.g., the balance between excitatory and inhibitory neurons in CROS models; Hardstone et al., 2014; Poil et al., 2012) and order parameters (e.g., active neuron density and synchronous degree; Dalla Porta & Copelli, 2019). A brain criticality hypothesis without strict definitions of control and order parameters is not informative (Cocchi et al., 2017; Girardi-Schappo, 2021). To present conceptual instances, we illustrate four possible critical phenomena in Figure 2, each of which corresponds to a concrete brain criticality type.

#### Instance of ordinary criticality.

To produce ordinary criticality (OC), we can control neural dynamics and manipulate 〈*θ*〉, the expectation of coupling strength *θ* among all neurons (e.g., averaged neural correlation), by some top-down and global biological effects. These effects, for instance, may be anesthesia effects (e.g., by ketamine-xylazine; Ribeiro et al., 2010; and isoflurane; Hahn et al., 2017) or sleep restoration effects; Meisel et al., 2013). We use the Kuramoto order parameter *ω* (Acebrón et al., 2005; Arenas et al., 2008) as the order parameter to define synchronous and asynchronous phases (di Santo et al., 2018; Fontenele et al., 2019). As 〈*θ*〉 increases, we may see transitions from asynchronous to synchronous phase in some situations (see a similar instance in Villegas, Moretti, & Muñoz, 2014). One can see Figure 2C for conceptual illustrations.

#### Instance of quasi-criticality.

To produce quasi-criticality (qC), we can manipulate refractory period length *τ*, branching ratio *κ*, and stimulus intensity *υ* as control parameters (e.g., control *τ* and *κ* by pharmacological perfusion or ionic concentration adjustment; Chiappalone et al., 2003; Shew et al., 2011). There exist a disordered phase (subcritical), an ordered but asynchronous phase (supercritical), and a synchronous (quasi-periodic) phase in the space of (*υ*, *τ*, *κ*) (Fosque et al., 2021; Williams-García et al., 2014). These phases can be characterized by specific order parameters related to synchronization. As *υ* increases, a qC phenomenon emerges in the space, where the quasi-critical region is defined by all combinations of (*υ*, *τ*, *κ*) whose susceptibility values are at least half-maximum. Cross-over behaviors (i.e., a generalization of phase transition with finite susceptibility) emerge when the quasi-critical region has overlaps with at least two phases (Fosque et al., 2021; Williams-García et al., 2014). In Figure 2D, we show this qC phenomenon in details.

#### Instance of self-organized criticality.

To study self-organized criticality (SOC), we consider the conserved neural dynamics generated by integrate-and-fire neurons (Levina et al., 2007). The order parameter is active neuron density *ρ*, whose dynamics is controlled by parameter 〈*θ*〉_*A*_, the averaged coupling strength *θ* between activated neurons and their postsynaptic neurons (here *A* denotes the set of activated neurons). In specific cases, the considered neural dynamics may self-organize to the critical point under the joint effects of excitatory and inhibitory neurons, neural spiking processes (activation and silence), as well as neural plasticity. In Figure 2E, we conceptually illustrate a case where these endogenous mechanisms enable the brain to self-organize to the criticality between absorbing and active phases.

#### Instance of self-organized quasi-criticality.

To analyze self-organized quasi-criticality (SOqC), we consider the nonconserved neural dynamics affected by two homeostatic adaptation processes, that is, the short-term depression of inhibition and the spike-dependent threshold increase. These processes are controlled by $\hat{y}$, the maximum inhibitory coupling strength, as well as *τ*_*x*_ and *τ*_*y*_, the decay timescales of neural activation threshold increase and synaptic depression. These control parameters affect neural activation threshold *x* and inhibition strength *y* to shape neural dynamics states (e.g., the active neuron density *ρ*). With appropriate *x*, *y*, and *ρ*, neural avalanches with power law behaviors will occur to indicate the criticality between an asynchronous phase (stochastic oscillations) and a synchronous phase (periodic oscillations). According to Girardi-Schappo et al. (2021), *x* and *ρ* self-organize to their appropriate values through quasi-critical fluctuations under biologically reasonable conditions (i.e., *τ*_*x*_ ≫ 1) while *y* hovers around the expected value. Additional fine tuning of *y* based on exogenous mechanisms are necessary to place neural dynamics at the perfect criticality. Meanwhile, synaptic homeostasis is discovered as constantly canceled by the variation of the activation threshold, impeding neural dynamics from self-organizing to perfect criticality. In Figure 2F, we conceptually illustrate the defined SOqC phenomenon in a similar manner of Figure 2D and Figure 2E. As for the precise description of quasi-critical fluctuations, one can see Girardi-Schappo et al. (2021) for details.

To this point, we have conceptually introduced the phenomenological properties of brain criticality. To verify the hypothetical brain criticality, one needs to learn about analytic brain criticality theories and the properties of neural avalanche predicted by them. Below, we present accessible expositions of these theoretical foundations.

## BRAIN CRITICALITY: PHYSICAL THEORIES

### Mean-Field and Stochastic Field Theories of Brain Criticality

One of the main challenges faced by neuroscientists in studying ordinary criticality (OC), quasi-criticality (qC), self-organized criticality (SOC), and self-organized quasi-criticality (SOqC) is how to understand their theoretical relations (Girardi-Schappo, 2021). Overcoming this challenge is crucial for understanding why we can verify the existence of different types of brain criticality with certain theoretical tools. To present a concise and thorough review, we first focus on brain criticality between absorbing and active phases, where we generalize the ideas in Bonachela and Muñoz (2009) and Buendía, di Santo, Bonachela, and Muñoz (2020a) to present a possible framework for unification.

#### Langevin formulation of ordinary criticality.

In general, brain criticality in the space of absorbing and active phases are related to directed percolation (Dalla Porta & Copelli, 2019), a universality class of continuous phase transitions into absorbing states (Hinrichsen, 2000; Lübeck, 2004). Here, a universality class can be understood as the set of all systems with the same scaling properties (Hinrichsen, 2000; Lübeck, 2004; Sethna, Dahmen, & Myers, 2001). Directed percolation theory initially covers OC phenomena (Hinrichsen, 2000; Lübeck, 2004). Let us begin with a variant of the classic Reggeon field theory, the simplest description of absorbing phase transitions (Henkel, Hinrichsen, Lübeck, & Pleimling, 2008). The Langevin equation of the activity neuron field *ρ*($\vec{x}$, *t*) is defined as

$$\frac{\partial }{\partial t}\rho \left(\vec{x},t\right)=\left(a+b\nu \left(\vec{x},t\right)\right)\rho \left(\vec{x},t\right)-c\rho^{2}\left(\vec{x},t\right)+d\nabla^{2}\rho \left(\vec{x},t\right)+e\sqrt{\rho \left(\vec{x},t\right)}\sigma \left(\vec{x},t\right),$$ (3)

$$\frac{\partial }{\partial t}\nu \left(\vec{x},t\right)=\nabla^{2}\nu \left(\vec{x},t\right)+f\left(\vec{x},t\right)-g\left(\vec{x},t\right)\rho \left(\vec{x},t\right),$$ (4)

where $\vec{x}$ represents spatial coordinates, *a* ∈ ℝ, *b* ∈ (0, ∞), *c* ∈ (0, ∞), *d* ∈ ℝ is the diffusion factor, and *e* ∈ ℝ is the noise factor. Function *σ*(·, ·) defines a zero-mean Gaussian noise with a spatiotemporal correlation 〈*ρ*($\vec{x}$, *t*) *ρ*($\vec{x}$′, *t*′)〉 = *δ*($\vec{x}$ − $\vec{x}$′) *δ*(*t* − *t*′), where *δ*(·) is the delta function. In general, *σ*(·, ·) reflects the collective fluctuations in neural activities that vanish in the absorbing phase *ρ*($\vec{x}$, *t*) = 0 under the effects of factor $\sqrt{\rho \left(\vec{x},t\right)}$. The term ∇^2^*ρ*($\vec{x}$, *t*) reflects the propagation of neural dynamics. The function *ν*($\vec{x}$, *t*) defines the energy (i.e., membrane potential) that propagates according to ∇^2^*ν*($\vec{x}$, *t*), increases with external drives *f*($\vec{x}$, *t*), and decreases with bulk dissipation *g*($\vec{x}$, *t*). Please note that *ρ*($\vec{x}$, *t*) ≥ 0 and *ν*($\vec{x}$, *t*) ≥ 0 always hold. The initial active neuron density and energy are assumed as nonzero. It is clear that *a* + *bν*($\vec{x}$, *t*) < 0 makes the neural dynamics eventually vanish (i.e., absorbing phase) while *a* + *bν*($\vec{x}$, *t*) > 0 does not (i.e., active phase). Therefore, we can fine tune the control parameter *ν*($\vec{x}$, *t*) to make the brain exhibit OC dynamics at *a* + *bν*_*c*_($\vec{x}$, *t*) = 0, a critical point defined by *ν*_*c*_. The fine tuning relies on manipulating *f*($\vec{x}$, *t*) and *g*($\vec{x}$, *t*) by exogenous mechanisms.

#### Langevin formulation of quasi-criticality.

Then we turn to analyzing qC, whose mean-field approximation is initially derived based on the cortical branching model (Fosque et al., 2021; Williams-García et al., 2014). A cortical branching model with no stimulus input belongs to the directed percolation universality class according to the Janssen-Grassberger conjecture (Williams-García et al., 2014). Nonzero stimulus inputs make the cortical branching model depart from directed percolation universality class to create qC (Williams-García et al., 2014). Nevertheless, the above mean-field theory is defined in the space of synchronous and asynchronous phases. To derive a qC phenomenon between absorbing and active phases, we can provisionally analyze a mean-field approximation of Equations 3 and 4:

$$\frac{\partial }{\partial t}\rho \left(\vec{x},t\right)=\left(a+b\nu \left(\vec{x},t\right)\right)\rho \left(\vec{x},t\right)-c\rho^{2}\left(\vec{x},t\right),$$ (5)

$$\frac{\partial }{\partial t}\nu \left(\vec{x},t\right)=f\left(\vec{x},t\right)-g\left(\vec{x},t\right)\rho \left(\vec{x},t\right),$$ (6)

where ∇^2^*ρ*($\vec{x}$, *t*), ∇^2^*ν*($\vec{x}$, *t*), and *σ*($\vec{x}$, *t*) in Equations 3 and 4 are neglected under the mean-field assumption. We consider the cases where stimulus inputs vanish, that is, *f*($\vec{x}$, *t*) ≡ 0. The critical point between active and absorbing phase becomes *ν*_*c*_ = −$\frac{a}{b}$. The steady state solutions of Equations 5 and 6 are

$$\rho \left(\vec{x},t\right)=0,$$ (7)

$$\nu \left(\vec{x},t\right)=r\in \left(0,\infty \right),$$ (8)

respectively. Therefore, OC is one of the steady states of neural dynamics when there is no stimulus. In the cases where stimulus inputs become increasingly strong, there exists no steady-state solution of Equations 5 and 6 unless $\frac{f\left(\vec{x},t\right)}{g\left(\vec{x},t\right)}$ → *r* ∈ (0, ∞). If $\frac{f\left(\vec{x},t\right)}{g\left(\vec{x},t\right)}$ → *r* ∈ (0, ∞) holds, we can derive

$$\rho \left(\vec{x},t\right)=\frac{f\left(\vec{x},t\right)}{g\left(\vec{x},t\right)}\to r,$$ (9)

$$\nu \left(\vec{x},t\right)=\frac{1}{b}\left(c\frac{f\left(\vec{x},t\right)}{g\left(\vec{x},t\right)}-a\right)\to \frac{1}{b}\left(cr-a\right).$$ (10)

Because the critical point *ν*_*c*_ = −$\frac{a}{b}$ is not necessarily a steady state, it can be disturbed by diverse factors (e.g., by stimuli). Unless there exist certain ideal exogenous mechanisms that persistently enlarge *g*($\vec{x}$, *t*) whenever *f*($\vec{x}$, *t*) increases, the fine tuning of neural dynamics cannot cancel the effects of *f*($\vec{x}$, *t*). Consequently, the fine tuning process may only enable the brain to reach a quasi-critical region where the susceptibility of neural dynamics is relatively large. The initial OC vanishes and is replaced by qC.

#### Langevin formulation of self-organized criticality.

Although SOC is treated as a rather isolated concept after its first discovery in statistical physics (Bak, Tang, & Wiesenfeld, 1987), subsequent analyses demonstrate SOC as relevant with ordinary continuous phase transitions into infinitely many absorbing states (Dickman, Muñoz, Vespignani, & Zapperi, 2000; Dickman, Vespignani, & Zapperi, 1998; Narayan & Middleton, 1994; Sornette, Johansen, & Dornic, 1995). Specifically, SOC models can be subdivided into two families, which we refer to as external dynamics family (e.g., Bak-Sneppen model; Bak & Sneppen, 1993) and conserved field family (e.g., sandpile models such as Manna model; Manna, 1991; and Bak-Tang-Wiesenfeld model; Bak et al., 1987). The second family, being the main theoretical source of studying SOC in neural dynamics, corresponds to absorbing-state transitions since it can represent any system with conserved local dynamics and continuous transitions to absorbing states (Dickman et al., 2000; Lübeck, 2004). Although the universality class of the second family should be precisely referred to as conserved directed percolation, the explicit behaviors (e.g., avalanche exponents and scaling relations) of conserved directed percolation are similar to those of directed percolation in high-dimensional systems (e.g., neural dynamics) (Bonachela & Muñoz, 2008, 2009; Buendía et al., 2020a). Therefore, SOC and OC share some identification criteria in practice. To understand the connections between SOC and OC more precisely, we can consider the cases where *f*($\vec{x}$, *t*) → 0 such that $\frac{f\left(\vec{x},t\right)}{g\left(\vec{x},t\right)}$ → 0 (i.e., infinite separation of timescales). The steady-state solutions of Equations 5 and 6 become

$$\rho \left(\vec{x},t\right)=\frac{f\left(\vec{x},t\right)}{g\left(\vec{x},t\right)}\to 0,$$ (11)

$$\nu \left(\vec{x},t\right)=\frac{1}{b}\left(c\frac{f\left(\vec{x},t\right)}{g\left(\vec{x},t\right)}-a\right)\to \nu_{c},$$ (12)

respectively. Self-organization properties are reflected by the following processes: if the brain is in the absorbing phase because neural dynamics vanishes, that is, *ρ*($\vec{x}$, *t*) → 0, Equation 6 becomes $\frac{\partial }{\partial t}$ *ν* ($\vec{x}$, *t*) = *f*($\vec{x}$, *t*) to shift the brain toward the active phase; if the brain is in the active phase, Equation 6 becomes $\frac{\partial }{\partial t}$ *ν* ($\vec{x}$, *t*) ≃ −*g*($\vec{x}$, *t*) *ρ*($\vec{x}$, *t*) to reduce neural dynamics since *f*($\vec{x}$, *t*) ≪ *g*($\vec{x}$, *t*). These feedback control loops drive the brain to the critical point. One may be curious about why energy conservation, that is, *g*($\vec{x}$, *t*) → 0, is necessary for SOC since the above derivations seem to be independent of *g*($\vec{x}$, *t*) → 0. Later we show that the absence of *g*($\vec{x}$, *t*) → 0 in Equation 14 makes the active phase no longer exist. In other words, the nonconserved energy implies a kind of continuous phase transition that does not belong to conserved directed percolation or directed percolation when the infinite separation of timescales is satisfied. Therefore, energy conservation is necessary for SOC.

#### Langevin formulation of self-organized quasi-criticality.

As for SOqC, nonzero bulk dissipation breaks the conservation law to generate non-Markovian components in neural dynamics (Bonachela & Muñoz, 2009; Buendía et al., 2020a). In the ideal cases where the drive terms (e.g., stimulus inputs) of a sufficiently large neural dynamics system occur at an arbitrarily slow timescale (i.e., only occur in the interval between neural avalanches), the brain exhibits pure dynamical percolation behaviors (Buendía et al., 2020a). To understand this property, let us consider a variant of Equations 5 and 6 where the dissipation term *g*($\vec{x}$, *t*) is nonnegligible

$$\frac{\partial }{\partial t}\rho \left(\vec{x},t\right)=\left(a+b\nu \left(\vec{x},t\right)\right)\rho \left(\vec{x},t\right)-c\rho^{2}\left(\vec{x},t\right),$$ (13)

$$\frac{\partial }{\partial t}\nu \left(\vec{x},t\right)=-g\left(\vec{x},t\right)\rho \left(\vec{x},t\right).$$ (14)

By integrating Equation 14 and plugging the integral into Equation 13, we can derive

$$\frac{\partial }{\partial t}\rho \left(\vec{x},t\right)=\left(a+b\nu \left(\vec{x},0\right)\right)\rho \left(\vec{x},t\right)-c\rho^{2}\left(\vec{x},t\right)-b\rho \left(\vec{x},t\right)\int_{0}^{t}g\left(\vec{x},t\right)\rho \left(\vec{x},\tau \right)d\tau ,$$ (15)

The non-Markovian term −*bρ*($\vec{x}$, *t*) $\int_{0}^{t}$*g*($\vec{x}$, *t*) *ρ*($\vec{x}$, *τ*)d*τ* in Equation 15 makes the regions already visited by neural dynamics become more unlikely to be activated (Bonachela & Muñoz, 2009; Buendía et al., 2020a). Therefore, the pure self-sustained active phase vanishes and is replaced by a spreading phase, where local perturbations can transiently propagate across the whole system without reaching a self-sustained state, and a nonspreading phase, where local perturbations can never span the entire system (Bonachela & Muñoz, 2009; Buendía et al., 2020a). The phase transition and corresponding critical point *ν*_*d*_ > *ν*_*c*_ between spreading and nonspreading phases belong to the universality class of dynamical percolation rather than conserved directed percolation (Bonachela & Muñoz, 2009; Buendía et al., 2020a). The initial neural dynamics can be created by random shifts at moment 0 (Bonachela & Muñoz, 2009; Buendía et al., 2020a)

$$\rho \left({\vec{x}}^{*},0\right)\to \epsilon ,$$ (16)

$$\nu \left({\vec{x}}^{*},0\right)\to \nu \left({\vec{x}}^{*},0\right)+h\left({\vec{x}}^{*},0\right),$$ (17)

where $\vec{x}$* is a randomly selected coordinate, and function *h*(·, ·) is a driving function of energy at moment 0. Every time a neural avalanche occurs after random shifts, the strong dissipation term *g*($\vec{x}$, *t*) pushes the brain towards the subcritical phase. Consequently, the brain cannot exactly self-organize to the perfect criticality. Instead, the brain just hovers around the critical point *ν*_*d*_ to form a quasi-critical region, exhibiting finite fluctuations to the both sides of *ν*_*d*_. In the more realistic cases where the drive terms do not necessarily occur at an arbitrarily slow timescale (i.e., can occur at an arbitrary moment), however, neural dynamics may be phenomenology controlled by conserved directed percolation transitions and hover around the critical point. Let us add a drive term in Equation 14

$$\frac{\partial }{\partial t}\nu \left(\vec{x},t\right)=f\left(\vec{x},t\right)-g\left(\vec{x},t\right)\rho \left(\vec{x},t\right).$$ (18)

Then Equation 15 becomes

$$\frac{\partial }{\partial t}\rho \left(\vec{x},t\right)=\left(a+b\nu \left(\vec{x},0\right)\right)\rho \left(\vec{x},t\right)-c\rho^{2}\left(\vec{x},t\right)-b\rho \left(\vec{x},t\right)\int_{0}^{t}\left(f\left(\vec{x},\tau \right)-g\left(\vec{x},\tau \right)\rho \left(\vec{x},\tau \right)\right)d\tau .$$ (19)

If we can ideally fine tune the drive term *f*($\vec{x}$, *t*) to ensure that $\frac{f\left(\vec{x},t\right)}{g\left(\vec{x},t\right)}$ → *r* ∈ (0, ∞), the steady-state solutions of Equations 18 and 19 are

$$\rho \left(\vec{x},t\right)=\frac{f\left(\vec{x},t\right)}{g\left(\vec{x},t\right)}\to r,$$ (20)

$$\nu \left(\vec{x},0\right)=\frac{1}{b}\left(c\frac{f\left(\vec{x},t\right)}{g\left(\vec{x},t\right)}-a\right)\to \frac{1}{b}\left(cr-a\right),$$ (21)

$$\nu \left(\vec{x},t\right)=\nu \left(\vec{x},0\right)+\int_{0}^{t}\left(f\left(\vec{x},\tau \right)-g\left(\vec{x},\tau \right)\rho \left(\vec{x},\tau \right)\right)d\tau \to \frac{1}{b}\left(cr-a\right).$$ (22)

Equations 20–22 correspond to a steady state of the brain with *ρ*($\vec{x}$, *t*) → *r* and conserved energy, which is similar to SOC. Therefore, the brain may self-organize to a quasi-critical region around *ν*_*c*_, the critical point of SOC. Reaching the critical point requires ideal fine tuning. These emerged conserved directed percolation behaviors enable scientists to recognize SOqC in a similar manner of SOC in practice (i.e., when stimulus inputs can occur at any moment) (Bonachela & Muñoz, 2009; Buendía et al., 2020a).

#### Summary of theoretical relations.

Taken together, neuroscientists can approximately verify the existence of brain criticality in the space of absorbing and active phases with specific tools coming from directed percolation theory. This is because OC, qC, SOC, and SOqC exhibit or approximately exhibit directed percolation behaviors under certain conditions. The verification may be inaccurate since the approximation holds conditionally. As for the brain criticality between asynchronous and synchronous phases, however, the universality class properties become rather elusive because an analytic and complete theory of synchronous phase transitions in the brain remains absent yet (see Buendía, Villegas, Burioni, & Muñoz, 2021; di Santo et al., 2018; for early attempts). Although some behaviors of absorbing phase transitions can be observed in synchronous phase transitions (e.g., see Buendía et al., 2021; di Santo et al., 2018; Fontenele et al., 2019; Girardi-Schappo et al., 2021), there also exist numerous differences between them (e.g., see Buendía et al., 2021; Fontenele et al., 2019; Girardi-Schappo et al., 2021). As suggested by Dalla Porta and Copelli (2019), it remains elusive if directed percolation properties are applicable, at least conditionally applicable, to analyzing synchronous phase transitions. More explorations are necessary in the future.

There are numerous properties of brain criticality predicted by directed percolation theory, among which, neural avalanche exponents (the power law exponents of lifetime and size distributions), scaling relation, universal collapse shape, and slow decay of autocorrelation are applicable in both analytic derivations and statistical estimations from empirical data. These properties are our main focus. For convenience, we summarize important glossaries and symbol conventions before we discuss theoretical details (Table 2).

**Table 2.** Glossaries and symbol conventions

Variable	Meaning
*T*	The lifetime of the neural avalanche
*S*	The size of the neural avalanche
*A*	The area of the neural avalanche
〈*S*(*T*)〉	The averaged size of neural avalanches with lifetime *T*
〈*S*(*t*|*T*)〉	The averaged time-dependent avalanche size at moment *t* during neural avalanches with the lifetime *T*
𝒫_*T*_(*t*)	The probability distribution of neural avalanche lifetime
𝒫_*S*_(*s*)	The probability distribution of neural avalanche size
*α*	Power law exponent of the neural avalanche lifetime distribution 𝒫_*T*_(*t*) ∝ *t*^−*α*^
*β*	Power law exponent of the neural avalanche size distribution 𝒫_*S*_(*s*) ∝ *s*^−*β*^
*γ*	Power exponent of the neural avalanche area *A* ∝ *T*^*γ*^
𝓗(·)	Universal scaling function
Cov(·, ·)	Autocorrelation function
*χ*	Power law decay rate of autocorrelation
*ξ*	Exponential decay rate of autocorrelation

*Note*. Please note that Table 2 mainly contains important glossaries with fixed symbol definitions. There are many symbols uncovered by Table 2 since they are only used for mathematical derivations.

### Neural Avalanche Exponents

As we have mentioned above, neural avalanches are expected to exhibit power law properties in their lifetime and size distributions when the brain is at the critical point (Hinrichsen, 2000; Larremore et al., 2012; Lübeck, 2004). Therefore, it is pivotal to confirm the detailed values of neural avalanche exponents. To analytically derive these exponents, one can consider critical branching process (di Santo, Villegas, Burioni, & Muñoz, 2017; García-Pelayo, Salazar, & Schieve, 1993; Gros, 2010; Harris, 1963; Otter, 1949), neural field theory (Robinson, 2021), mean-field Abelian sandpile models (Janowsky & Laberge, 1993; D. S. Lee, Goh, Kahng, & Kim, 2004), and avalanches in networks (Larremore et al., 2012). The key idea to derive neural avalanche exponents shared by these existing theories is to confirm the explicit forms of 𝒫_*T*_(*t*) and 𝒫_*S*_(*s*), the probability distributions of the lifetime and size of neural avalanches, under ideal conditions (e.g., when the maximum lifetime and size are unlimited and can be infinitely large). In real cases where lifetime and size are restricted because the brain is a finite system, slight deviations from idea values may be observed but theoretical derivations of neural avalanche exponents principally hold.

To present accessible expositions, we consider a critical branching process in Equations 23–34 to describe related backgrounds. More importantly, we present a novel and simple idea to calculate target exponents in the context of neuroscience in Box 1. Abstractly, one can define 𝒫(*n*, *t*) as the probability for an active neuron at moment *t* to activate *n* postsynaptic neurons subsequently and define 𝒵(*n*, *t*) as the probability of finding *n* active neurons at moment *t*. Meanwhile, one denotes

$$𝓕\left(x,t\right)=\sum_{n=0}^{\infty }𝒫\left(n,t\right)x^{n},$$ (23)

$$𝒢\left(x,t\right)=\sum_{n=0}^{\infty }𝒵\left(n,t\right)x^{n}$$ (24)

as the corresponding generating functions (Fristedt & Gray, 2013; Rao & Swift, 2006). Then, one can readily see the recursion relation

$$𝒢\left(x,t\right)=\sum_{n=0}^{\infty }𝒵\left(n,t-\delta t\right)𝓕{\left(x,t-\delta t\right)}^{n},$$ (25)

$$\;=𝒢\left(𝓕\left(x,t-\delta t\right),t-\delta t\right),$$ (26)

where *δt* denotes the minimum time step. Equation 26 implies that branching processes are Markovian. Similarly, one can measure the expectations

$$\mu \left(t\right)={\left.\frac{\partial }{\partial x}𝓕\left(x,t\right)\right|}_{x=1},$$ (27)

$$\phi \left(t\right)={\left.\frac{\partial }{\partial x}𝒢\left(x,t\right)\right|}_{x=1}$$ (28)

to derive another recursion relation

$$\phi \left(t\right)={\left.\frac{\partial }{\partial x}𝓕\left(x,t-1\right)\right|}_{x=1}{\left.\frac{\partial }{\partial x}𝒢\left(x,t-1\right)\right|}_{x=1},$$ (29)

$$\;=\phi \left(t-\delta \right)\mu \left(t-\delta \right),$$ (30)

$$\;=\prod_{\tau =0}^{t-\delta }\mu \left(\tau \right).$$ (31)

Note that Equation 31 is derived from the fact that *ϕ*(0) = 1 (one neuron is activated at moment 0 to trigger neural avalanches). Please see Marković and Gros (2014) for more explanations of Equations 25–31. Assuming that *ϕ*(*t*) scales as exp(*λt*) for large *t*, we know that *ϕ*(*t*) converges to 0 given a negative Lyapunov exponent *λ* (the branching process is subcritical; di Santo et al., 2017; García-Pelayo et al., 1993; Gros, 2010; Harris, 1963; Otter, 1949) and diverges with a positive Lyapunov exponent *λ* (the branching process is supercritical; di Santo et al., 2017; García-Pelayo et al., 1993; Gros, 2010; Harris, 1963; Otter, 1949). Here *λ* can be defined according to Equation 32

$$\lambda =\lim_{t\to \infty }\;\ln\left(\frac{1}{t}\phi \left(t\right)\right)=\lim_{t\to \infty }\frac{1}{t}\sum_{\tau =0}^{t-\delta }\ln\left(\mu \left(\tau \right)\right).$$ (32)

If the branching process is homogeneous, namely 𝒫(*n*, *t*) = 𝒫(*n*), 𝒵(*n*, *t*) = 𝒵(*n*), *μ*(*τ*) = *μ*, and *ϕ*(*τ*) = *ϕ* for every moment *τ*, then *μ* = 1 is the condition for the branching process to be critical. To relate these results with neural avalanches, one only need to consider the avalanche size *S* = ∑_*t*_ *z*(*t*), where *z*(*t*) ∼ 𝒵 denotes the number of active neurons at moment *t*, and the avalanche life time *T* = min{*t*|*z*(*t*) > 0 and *z*(*t* + *δt*) = 0}. It has been analytically proved that in terms of fixed environments and a Poisson generating function 𝓕 one can derive (Otter, 1949)

$${𝒫}_{S}\left(s\right)∼s^{-3/2}\mu^{s-1}\exp\left[s\left(1-\mu \right)\right],$$ (33)

$${𝒫}_{T}\left(t\right)∼t^{-2}\mu^{t-1}\exp\left[t\left(1-\mu \right)\right].$$ (34)

In the case with *μ* = 1, one can obtain 𝒫_*S*_(*s*) ∼ *s*^−3/2^ and 𝒫_*T*_(*t*) ∼ *t*^−2^, the power law distributions of neural avalanche size and neural avalanche life time (di Santo et al., 2017; García-Pelayo et al., 1993; Gros, 2010; Harris, 1963; Janowsky & Laberge, 1993; Jung, Le, Lee, & Lee, 2020; Larremore et al., 2012; D. S. Lee et al., 2004; Lombardi, Herrmann, & De Arcangelis, 2017; Otter, 1949; Robinson, 2021), from Equations 33 and 34.

**Figure 3.**
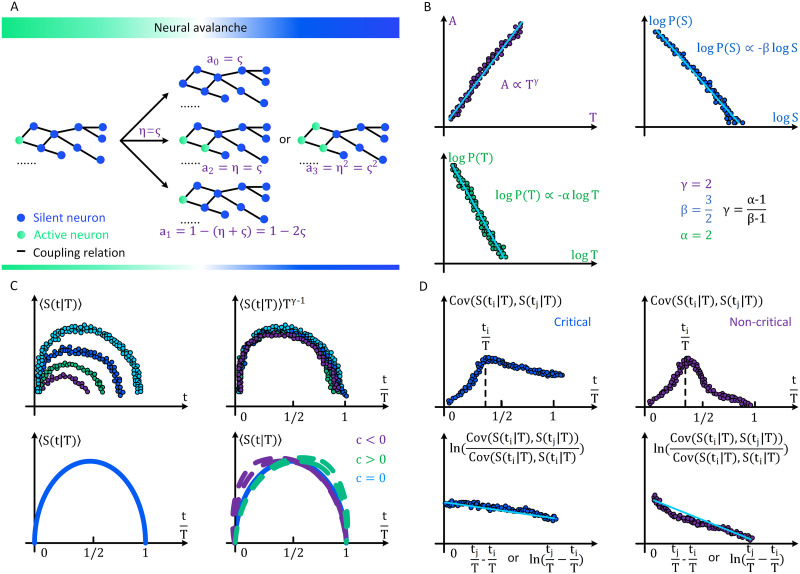
Conceptual illustrations of the neural avalanche properties predicted by analytic theories of brain criticality. (A) Illustrations of the framework to derive neural avalanche exponents in Box 3. (B) Illustrations of the scaling relation satisfied by neural avalanches under mean-field assumptions. (C) Illustrations of the universal collapse shape of neural avalanches. The unscaled plot of *t* versus 〈*S*(*t*|*T*)〉 (upper left) and the scaled plot 〈*S*(*t*|*T*)〉*T*^1−*γ*^ versus $\frac{t}{T}$ (upper right) are shown for comparison. Here terms $\frac{1}{T}$ and *T*^1−*γ*^ respectively serve as scale factors on *x*-axis and *y*-axis to create a universal collapse shape. Meanwhile, the symmetric collapse shape in Equation 51 (bottom left) and the asymmetric collapse shape controlled by skewness *c* in Equation 52 (bottom right) are also presented. (D) Autocorrelations and their decays in critical (left) and noncritical (right) cases are shown. Autocorrelations are calculated after a *t*_*i*_ ∈ [0, *T*) is randomly selected as a reference (upper left and right). Meanwhile, the autocorrelation decays measured on *t*_*j*_ ∈ [*t*_*i*_, *T*] is fitted to derive the coefficients *χ* (*x*-axis corresponds to ln($\frac{t_{j}-t_{i}}{T}$)) and *ξ* (*x*-axis corresponds to $\frac{t_{j}-t_{i}}{T}$) in Equation 53 and Equation 55 (bottom left and right). One can see that autocorrelations in the critical case have slower decays (smaller *χ* and *ξ*) than those in the noncritical case.

The derivations of avalanche exponents *α* = 2 and *β* = $\frac{3}{2}$ are nontrivial. However, few neuroscience studies elaborate on these details, impeding researchers from understanding the theoretical foundations of brain criticality in the brain. The importance of these derivations is beyond the detailed values of avalanche exponents since they reveal the fundamental properties of neural dynamics (Cocchi et al., 2017; di Santo et al., 2017; Girardi-Schappo, 2021). In Box 1, we sketch an original idea to derive these avalanche exponents in the terminology of neuroscience. In Figure 3A, we present graphical illustrations of our idea in Box 1.

**Box 1.** Derivations of neural avalanche exponents Consider a time-continuous neural dynamics process, where an active neuron implies three possibilities: becoming absorbed with probability *ς*, activating another neuron with probability *η*, or remaining effect-free with probability 1 − (*ς* + *η*). In critical states, we have *ς* = *η* (García-Pelayo et al., 1993). We define 𝒜_*n*_(*t*) as the probability for *n* active neurons to exist at t* + *t* given that 1 active neuron exists at t*. Assuming the independence of neuron activation, we have

$${𝒜}_{n}\left(t\right)=\sum_{n_{1}+\ldots +n_{k}=n}{𝒜}_{n_{1}}\left(t\right)\ldots {𝒜}_{n_{k}}\left(t\right).$$ (35)

If 𝒜_*n*_(*t*), *n* ∈ ℕ^+^ admits a Maclaurin expansion 𝒜_*n*_(*t*) = *a*_*n*_*t* + *o*(*t*^2^) (when *n* ≠ 1) or 𝒜_*n*_(*t*) = *a*_*n*_*t* + 1 + *o*(*t*^2^) (when *n* = 1) where *a*_*n*_ = d𝒜_*n*_(0)/d*t*, we can readily derive *a*_0_ = *a*_2_ = *ς* and *a*_1_ = −2*ς* (García-Pelayo et al., 1993). Meanwhile, we can know

$${𝒜}_{n}\left(t+dt\right)-{𝒜}_{n}\left(t\right)=\sum_{k=0}^{\infty }a_{k}{𝒜}_{n-k}\left(t\right)dt.$$ (36)

Equations 15 and 16 readily lead to

$$\frac{\partial }{\partial t}𝒲\left(x,t\right)=\sum_{k=0}^{\infty }a_{k}\sum_{n=0}^{\infty }\left(\sum_{n_{1}+\ldots +n_{k}=n-k}\prod_{i=1}^{k}{𝒜}_{n_{i}}\left(t\right)\right)x^{n}=\sum_{k=0}^{\infty }a_{k}𝒲{\left(x,t\right)}^{k},$$ (37)

where 𝒲(*x*, *t*) = $\sum_{n=0}^{\infty }$ 𝒜_*n*_(*t*)*x*^*n*^, *x* ∈ [0, 1] denotes the generating function. Applying a trick introduced in García-Pelayo et al. (1993), we define 𝓗(*x*) = $\frac{\partial }{\partial t}$𝒲(*x*, 0), which naturally leads to $\frac{\partial }{\partial t}$𝒲(*x*, *t*) = 𝓗(𝒲(*x*, *t*)). Meanwhile, 𝓗(*x*) = *ς*(1 − *x*)^2^ can be derived based on *a*_0_, *a*_1_, and *a*_2_ (García-Pelayo et al., 1993). Taken together, we have

$$\frac{\partial }{\partial t}𝒲\left(x,t\right)=\varsigma {\left(1-𝒲\left(x,t\right)\right)}^{2}.$$ (38)

Note that the initial condition is 𝒲(*x*, 0) = *x* since one neuron is activated at t*. Solving Equation 38, we derive that

$$𝒲\left(x,t\right)=\frac{\varsigma \left(1-x\right)t}{\varsigma \left(1-x\right)t+1}.$$ (39)

Therefore, we have 𝒜_0_(*t*) = 𝒲(0, *t*) = $\frac{\varsigma t}{\varsigma t+1}$, supporting a calculation of lifetime distribution 𝒫_*T*_(*t*)

$$\lim_{t\to \infty }{𝒫}_{T}\left(t\right)=\lim_{t\to \infty }\frac{d}{dt}𝒲\left(0,t\right)∼t^{-2}.$$ (40)

Following García-Pelayo et al. (1993), Harris (1963), and Otter (1949), one can similarly calculate

$$\lim_{s\to \infty }{𝒫}_{S}\left(s\right)∼s^{-\frac{3}{2}}.$$ (41)

There are three important things to remember. First, the lifetime exponent *α* = 2 and size exponent *β* = $\frac{3}{2}$ can only be treated as ideal exponents under mean-field assumptions of directed percolation. There are numerous factors, such as granularity, network topology, and neural dynamics variability, that can be considered in derivations to affect the detailed values of avalanche exponents (Bonachela & Muñoz, 2009; Girardi-Schappo, 2021). In Table 3, we summarize the possible intervals of *α* and *β* in empirical neural data. Second, *α* and *β* alone are not sufficient to verify the existence of brain criticality. Even when the actual values of *α* and *β* in empirical data are exactly equal to theoretical predictions, they may still not satisfy the scaling relation and universal collapse. Meanwhile, as we shall discuss later, estimating *α* and *β* in practice is statistically error prone. Third, one cannot confirm or disprove a detail type of brain criticality based on *α* and *β* unless additional information is provided. Although four types of brain criticality exhibit (e.g., OC) or approximately exhibit (e.g., qC, SOC, and SOqC) directed percolation behaviors under certain conditions, these preconditions are difficult to verify in practice.

**Table 3.** Neural avalanche exponents with scaling relation in empirical data

Data type	Observed interval of *α*	Observed interval of *β*	Observed interval of *γ*
LFP recordings filtered by spike sorting	*α* ∈ [1.35, 2.67]	*β* ∈ [1.3, 2.5]	*γ* ∈ [1.16, 1.48]
LFP recordings with thresholding	*α* ∈ [1.82, 2.84]	*β* ∈ [1.57, 2.59]	*γ* ∈ [1.12, 1.39]
Ca and voltage imaging	*α* ∈ [2.15, 3.5]	*β* ∈ [1.5, 2.3]	*γ* ∈ [1.75, 2.5]

*Note*. The data is acquired from Girardi-Schappo (2021), where 45 experimental observations of neuronal avalanches reported by 30 studies are summarized. These observations can be classified according to the recording techniques of neural avalanches. Detailed data classification criteria (e.g., details of spike sorting and thresholding) can be seen in Girardi-Schappo (2021). We only include the data where *α*, *β*, and *γ* are all recorded and satisfy the scaling relation in Equation 45. For LFP recordings filtered by spike sorting, included observations are reported by Carvalho et al. (2021), Fontenele et al. (2019), Fosque et al. (2021), Ma et al. (2019), Mariani et al. (2021), and Senzai et al. (2019). For LFP recordings with thresholding, included observations come from Mariani et al. (2021) and Shew et al. (2015). For Ca and voltage imaging, observations are provided by Ponce-Alvarez et al. (2018) and Yaghoubi et al. (2018). Although numerous studies report neural avalanches in whole-brain imaging (e.g., MEG, M/EEG, and invasive ECoG), these studies either do not report three exponents together (Palva et al., 2013; Shriki et al., 2013; Zhigalov et al., 2015) or have not observed the scaling relation in Equation 45 (Varley et al., 2020). One can see Girardi-Schappo (2021) for a summary of these results.

### Scaling Relation

In the previous section, we discuss how the neural avalanche lifetime and size distributions exhibit power law properties when the brain is at the critical point (Hinrichsen, 2000; Larremore et al., 2012; Lübeck, 2004). Apart from lifetime *T* and size *S*, there are several other quantities that characterize neural avalanches, such as area *A* (number of distinct active neurons, measured as *A* ≃ 〈*S*(*T*)〉 where the expectation 〈·〉 is averaged across all neural avalanches with the same lifetime *T*) and radius exponent *R* (radius of gyration) (Lübeck, 2004; Lübeck & Heger, 2003). In general, the corresponding probability distributions of these four quantities decay algebraically

$${𝒫}_{X}\left(x\right)\propto x^{-\lambda_{X}},$$ (42)

where random variable *X* ∈ {*S*, *T*, *A*, *R*} can be an arbitrary quantity to characterize neural avalanches. The avalanche exponent *λ*_*X*_ is defined according to the selected meaning of *X* (e.g., *λ*_*T*_ = 2 and *λ*_*T*_ = $\frac{3}{2}$ under mean filed assumptions). Assuming that variables {*S*, *T*, *A*, *R*} scale as a power of each other

$$X^{\prime }\propto X^{\psi_{X^{\prime }X}},\forall X,X^{\prime }\in \left\{S,T,A,R\right\},$$ (43)

we can derive the scaling relation from Equations 42 and 43

$$\psi_{X^{\prime }X}=\frac{\lambda_{X}-1}{\lambda_{X^{\prime }}-1}.$$ (44)

If we let *X*′ = *A* and *X* = *T*, we can specify Equation 44 as

$$\gamma =\frac{\alpha -1}{\beta -1},$$ (45)

where 𝒫_*T*_(*t*) ∝ *t*^−*α*^, 𝒫_*S*_(*s*) ∝ *s*^−*β*^, and *A* ∝ *T*^*γ*^. Equation 45 leads to *γ* = 2 in the mean-field theory of directed percolation. In Table 3, one can see the possible interval of *γ* in empirical neural data. Equation 45 is widely used as a criterion to verify if the brain is at the critical point in neuroscience studies (e.g., Dalla Porta & Copelli, 2019; Fontenele et al., 2019; N. Friedman et al., 2012; Ponce-Alvarez et al., 2018). Once the scaling relation is confirmed among observed neural avalanche exponents, it indicates key features of the universality class (please note that *α*, *β*, and *γ* should be derived independently). For neuroscientists, the importance of Equation 45 lies in that it provides extra verification of the validity of estimated neural avalanche exponents. This verification is necessary given that neural avalanche exponent estimation is frequently inaccurate (Fontenele et al., 2019). In Figure 3B, we illustrate the scaling relation in Equation 45 under mean-field assumptions.

In Lübeck (2004), one can further learn about how brain criticality is mapped to a directed percolation transition characterized by ordinary critical exponents. Meanwhile, one can see how to connect these neural avalanche exponents with second-order phase transition exponents (Lübeck & Heger, 2003).

### Universal Collapse Shape

#### Universal collapse with an implicit scaling function.

Apart from the scaling relation discussed above, the average temporal shape of bursts, a fundamental signature of avalanches (Baldassarri, Colaiori, & Castellano, 2003; Laurson et al., 2013; Papanikolaou et al., 2011), can also be used to verify the existence of brain criticality in a more precise manner. This approach has been previously applied on diverse physical systems, such as plastically deforming crystals (Laurson & Alava, 2006) and Barkhausen noise (Mehta, Mills, Dahmen, & Sethna, 2002; Papanikolaou et al., 2011), and was recently introduced into neuroscience (Dalla Porta & Copelli, 2019; Fontenele et al., 2019; N. Friedman et al., 2012; Pausch, Garcia-Millan, & Pruessner, 2020); Ponce-Alvarez et al., 2018). To understand this approach, let us step back to the power relation in Equation 43 and specify that *X*′ = *A* and *X* = *T*. These settings naturally lead to

$$A≃\langle S\left(T\right)\rangle \equiv \int_{0}^{T}\langle S\left(t|T\right)\rangle dt\propto T^{\gamma },$$ (46)

where 〈*S*(*t*|*T*)〉 measures the averaged time-dependent avalanche size during an avalanche and the expectation 〈·〉 is averaged across all neural avalanches with the same lifetime *T*. Equation 46 can be readily reformulated as

$$\langle S\left(t|T\right)\rangle \propto T^{\gamma -1}.$$ (47)

The general form of Equation 47 is usually given by Baldassarri et al. (2003), Laurson et al. (2013), and Papanikolaou et al. (2011)

$$\langle S\left(t|T\right)\rangle =T^{\gamma -1}𝓗\left(\frac{t}{T}\right),$$ (48)

where 𝓗(·) denotes a universal scaling function. When the brain is at the critical point, all data of 〈*S*(*t*|*T*)〉*T*^1−*γ*^ is expected to collapse onto 𝓗(·) with reasonable errors (Baldassarri et al., 2003; Laurson et al., 2013; Papanikolaou et al., 2011). Here the terminology “collapse onto” means that all data generally exhibits a similar pattern in a plot of 〈*S*(*t*|*T*)〉*T*^1−*γ*^ vs. $\frac{t}{T}$ (e.g., all data follows function 𝓗(·)). Meanwhile, scaling function 𝓗(·) is expected to be a parabolic function (Baldassarri et al., 2003; Laurson et al., 2013; Papanikolaou et al., 2011). By testing these properties, neuroscientists can verify whether the brain is at criticality (e.g., Dalla Porta & Copelli, 2019; Fontenele et al., 2019; N. Friedman et al., 2012; Ponce-Alvarez et al., 2018). In Figure 3C, we graphically illustrate these properties.

#### Universal collapse with an explicit scaling function.

Under specific conditions, researchers can further consider an explicit form of scaling function 𝓗(·) (Laurson et al., 2013). Assuming that the early-time growth of neural avalanches averagely follows a power law of time, one can derive that 〈*S*(*t*|*T*)〉 ∝ *t*^*κ*^ for certain $\frac{t}{T}$ ≤ *ε* ≪ 1. Meanwhile, one knows that 〈*S*(*εT*|*T*)〉 ∝ *T*^*γ*−1^ should hold according to Equation 28. To ensure these two properties, one needs to have 〈*S*(*εT*|*T*)〉 ∝ (*εT*)^*κ*^ ∝ *T*^*γ*−1^, which readily leads to *κ* = *γ* − 1. Based on these derivations, one can know

$$\langle S\left(t|T\right)\rangle \propto t^{\gamma -1},\;t\ll T.$$ (49)

To find an explicit form of 𝓗(·) that satisfies Equations 48 and 49, one can consider a possible answer (Laurson et al., 2013)

$$𝓗\left(\frac{t}{T}\right)={\left[\frac{t}{T}\left(1-\frac{t}{T}\right)\right]}^{\gamma -1},$$ (50)

which can be analytically derived by multiplying Equation 49 by (1 − $\frac{t}{T}$)^*γ*−1^. Here (1 − $\frac{t}{T}$)^*γ*−1^ is a term to characterize the deceleration at the ends of neural avalanches (Laurson et al., 2013). Because *γ* = 2 is expected for critical neural avalanches under mean-field assumptions, Equations 48 and 50 imply that

$$\langle S\left(t|T\right)\rangle \propto t\left(1-\frac{t}{T}\right).$$ (51)

This result is consistent with the prediction by the ABBM model in the limit of vanishing drive rate and demagnetizing factor (N. Friedman et al., 2012; Laurson et al., 2013).

A potential limitation of Equation 51 in applications lies in its internal symmetry property (Laurson et al., 2013). Although avalanches under mean-field frameworks have a symmetric average shape (N. Friedman et al., 2012), it does not mean that symmetry generally holds in real complex systems (Laurson et al., 2013). Applying Equation 48 on neural data, researchers may observe a nonstandard parabolic function 𝓗(·) with specific skewness. This does not necessarily mean that neural dynamics is not at criticality. When neural avalanches are time irreversible (this is generally true in the brain since the detailed balance of neural dynamics is frequently broken; Lynn et al., 2021), one can consider small temporal asymmetry in the collapse shape (Laurson et al., 2013). To characterize potential asymmetry, one can add a correction term controlled by skewness degree *c* into Equation 51

$$\langle S\left(t|T\right)\rangle \propto t\left(1-\frac{t}{T}\right)\left[1-c\left(\frac{t}{T}-\frac{1}{2}\right)\right].$$ (52)

If *c* = 0, then Equation 52 reduces to Equation 51. Otherwise, neural avalanches can have a temporally asymmetric collapse shape with a positive (*c* > 0) or negative (*c* < 0) skewness (Laurson et al., 2013). We suggest that Equation 52 may be more applicable to real data of neural dynamics. In Figure 3C, we show examples of Equations 51 and 52.

### Slow Decay of Autocorrelation

In applications, researchers can also consider a more practical verification of the potential brain criticality. When the brain is at the critical point, a slow decay of autocorrelation is expected to occur in neural avalanches, corresponding to long-range correlations (Dalla Porta & Copelli, 2019; Erdos, Kruger, & Renfrew, 2018; Schaworonkow, Blythe, Kegeles, Curio, & Nikulin, 2015; Smit et al., 2011). This slow decay property is initially derived from the power law decay of autocorrelation, which can be analytically derived as a part of the scaling relation if ordinary critical exponents of directed percolation transition are considered (for details see Girardi-Schappo, 2021). The power law decay is expressed as

$$\ln\left[\frac{\text{Cov}\left(S\left(t_{i}|T\right),S\left(t_{j}|T\right)\right)}{\text{Cov}\left(S\left(t_{i}|T\right),S\left(t_{i}|T\right)\right)}\right]=-\chi \ln\left(\frac{t_{j}-t_{i}}{T}\right)+r,$$ (53)

where *t*_*i*_ ∈ [0, *T*) is used as a reference and *t*_*j*_ ∈ [*t*_*i*_, *T*] traverses the entire interval (Schaworonkow et al., 2015; Smit et al., 2011). According to the Wiener–Khinchin theorem, coefficient *χ* is related to 𝒮(*f*), the power spectrum of neural avalanches (notion *f* denotes frequency) (Bak et al., 1987; Girardi-Schappo, 2021; Linkenkaer-Hansen, Nikouline, Palva, & Ilmoniemi, 2001). One may expect 𝒮(*f*) ∼ *f*^−*υ*^ at the critical point, where *χ* = 1 − *υ* (Bak et al., 1987; Girardi-Schappo, 2021; Linkenkaer-Hansen et al., 2001). The power law decay of autocorrelation in Equation 53 breaks down when *υ* = 1, leading to infinitely long temporal correlations. Therefore, *χ* ∈ [0, ∞) in Equation 53 is expected to be sufficiently small. Certainly, the actual value of *χ* may not be perfectly zero in empirical data. For instance, *χ* ∈ [0.58 ± 0.23, 0.73 ± 0.31] and *χ* ∈ [0.52 ± 0.35, 0.81 ± 0.32] are observed in spontaneous alpha oscillations in MEG and EEG data, respectively (Linkenkaer-Hansen et al., 2001).

Apart from verifying power law decay directly, one can also consider the exponential decay, which is active in neuroscience as well (Miller & Wang, 2006; Pausch et al., 2020; Wilting & Priesemann, 2019). The exponential decay can described by

$$\frac{\partial }{\partial t}\text{Cov}\left(S\left(t_{i}|T\right),S\left(t_{j}|T\right)\right)=-\xi \;\text{Cov}\left(S\left(t_{i}|T\right),S\left(t_{j}|T\right)\right).$$ (54)

Equation 54 directly leads to

$$\ln\left[\frac{\text{Cov}\left(S\left(t_{i}|T\right),S\left(t_{j}|T\right)\right)}{\text{Cov}\left(S\left(t_{i}|T\right),S\left(t_{i}|T\right)\right)}\right]=-\xi \left(\frac{t_{j}-t_{i}}{T}\right)+r.$$ (55)

The exponential decay can be seen in the dynamics with short-term correlations (i.e., correlations have a characteristic time scale). Mathematically, the exponential decay can be related to power law decay in a form of *x*^−*y*^ = Γ(*y*) $\int_{0}^{\infty }$ *z*^*y*−1^ exp(−*xz*)d*z*, where Γ(·) denotes the Gamma function. When *ξ* ∈ [0, ∞) is sufficiently small, Equation 55 can be treated as a looser criterion that approximately verifies the slow decay of autocorrelation and may be more applicable to nonstandard brain criticality (e.g., qC and SOqC) (Wilting & Priesemann, 2019). Despite of its practicality, this looser criterion should be used with caution since it is not analytically derived from criticality theories.

In Figure 3D, we illustrate examples of autocorrelation slow decay in critical cases and compare them with noncritical cases. Compared with other properties previously discussed, a slow autocorrelation decay can be readily verified by conventional data fitting. However, we need to note that one should not confirm or reject the possibility of brain criticality only based on the decay characteristic of autocorrelation in Equations 53–55. This is because Equations 53–55 only serve as the approximate descriptions of long-range correlations at criticality. The strict criterion *χ*, *ξ* → 0 is rarely seen in empirical data while the determination of whether *χ* and *ξ* are sufficiently small in the looser criterion is relatively subjective.

In summary, we have reviewed the physical foundations of identifying and characterizing criticality in the brain. Based on these analytic derivations, we attempt to present systematic explanations of what is brain criticality and how to identify potential criticality in neural dynamics. Nevertheless, physical theories alone are not sufficient to support neuroscience studies because the implementation of these theories on empirical data is even more challenging than the theories themselves. To overcome these challenges, one needs to learn about statistic techniques to computationally estimate brain criticality from empirical data.

## BRAIN CRITICALITY: STATISTIC TECHNIQUES

While most properties of neural avalanches analytically predicted by the physical theories of brain criticality can be estimated by conventional statistic techniques, there exist several properties that frequently imply serious validity issues and deserve special attention. Below, we discuss them in detail.

### Estimating Neural Avalanche Exponents

Perhaps the estimation of neural avalanche exponents from empirical data is the most error-prone step in brain criticality analysis. The least-square approach is abused in fitting power law data and frequently derives highly inaccurate results (Clauset, Shalizi, & Newman, 2009; Virkar & Clauset, 2014). To derive neural avalanche exponents *α* and *β* in Equation 25 with reasonable errors, one needs to consider the maximum likelihood estimation (MLE) approach and corresponding statistic tests (see MLE on unbinned data; Clauset et al., 2009; and binned data; Virkar & Clauset, 2014). Taking the avalanche size distribution as an instance, the estimator $\hat{\beta }$ of distribution exponent *β* is expected to maximize the log-likelihood function

$$𝓛\left(\beta \right)=-n\ln\left[\zeta \left(\beta ,s^{\prime }\right)\right]-\beta \sum_{i=1}^{n}\ln\left(s_{i}\right),$$ (56)

$$𝓛\left(\beta \right)=n\left(\beta -1\right)\ln b^{\prime }+\sum_{i=1}^{k}h_{i}\ln\left(b_{i}^{1-\beta }-b_{i+1}^{1-\beta }\right).$$ (57)

Here Equation 56 and Equation 57 denote the log-likelihood functions on unbinned and binned data, respectively. Function *ζ*(·, ·) is the generalized zeta function (Bauke, 2007; Clauset et al., 2009). Notion *s* denotes avalanche size samples in Equation 33 and Equation 41 (Clauset et al., 2009). Notion *b* denotes bin boundaries defined on these samples and *h* counts the number of samples within each bin (Virkar & Clauset, 2014). Notions *s*′ and *b*′ are the lower cutoffs of unbinned and binned power law distributions (Clauset et al., 2009; Virkar & Clauset, 2014). They are necessary because few empirical data exhibits power law properties on the entire distribution (Clauset et al., 2009). Notions *n* and *k* measure the numbers of samples and bins above cutoffs, respectively (Clauset et al., 2009; Virkar & Clauset, 2014). To estimate $\hat{\beta }$ precisely, researchers are suggested to follow several indispensable steps (Clauset et al., 2009; Virkar & Clauset, 2014): (1) for each potential choice of *s*′ or *b*′, estimate the power law model on the distribution tail above the cutoff. Compute the Kolmogorov–Smirnov (KS) goodness-of-fit statistic between the cumulative probability distributions of power law model and empirical data. Find the ideal choice of *s*′ or *b*′ that minimizes KS statistic; (2) derive the corresponding estimator $\hat{\beta }$ and KS statistic based on the chosen cutoff; (3) use the semiparametric bootstrap to generate numerous synthetic data distributions that follow the estimated power law model above the cutoff but follow the empirical data distribution below the cutoff. Estimate new power law models on these synthetic data distributions and measure the goodness-of-fit by KS statistic. Define a *p* value, the fraction of these KS statistics in step 3 that are no less than the KS statistic in step 2. Rule out the estimated power law model in steps 1–2 if *p* < 0.1 (conservative criterion). Apart from these necessary steps, one can further consider Vuong’s likelihood ratio test for alternative distribution checking (Clauset et al., 2009; Virkar & Clauset, 2014; Vuong, 1989) and information loss measurement of binning approach (Virkar & Clauset, 2014). During the above process, we measure the goodness-of-fit by KS statistic instead of the well-known *χ*^2^ statistic because the latter has less statistic power (Bauke, 2007; Clauset et al., 2009; Virkar & Clauset, 2014). Meanwhile, KS statistic is measured on cumulative probability distributions rather than probability distributions to control the effects of extreme values in empirical data (Clauset et al., 2009; Virkar & Clauset, 2014). Except for the above approach (Clauset et al., 2009; Virkar & Clauset, 2014), one can also consider the BIC method (for unbinned data) (Schwarz, 1978) and the RT method (for binned data) (Reiss & Thomas, 2007) for comparisons. In practice, the approaches proposed by Clauset et al. are more robust (Clauset et al., 2009; Virkar & Clauset, 2014) and have attracted numerous follow-up studies for improvements (e.g., Deluca & Corral, 2013; Marshall et al., 2016; Yu, Klaus, Yang, & Plenz, 2014).

### Estimating Universal Collapse Shape

Another error-prone step is the calculation and evaluation of the universal collapse shape, which is closely related to scaling relation analysis. Deriving the collapse shape from empirical data may be problematic because the goodness evaluation of collapse shape is rather subjective (e.g., depends on personal opinions about whether all data follows function 𝓗(·) in Equation 48) in most cases (Marshall et al., 2016). Although important efforts have been devoted to quantify if a given dataset exhibits shape collapse (Bhattacharjee & Seno, 2001; Shaukat & Thivierge, 2016), common approaches in practice still depend on specific shape collapse algorithms that search potential scaling parameters (e.g., *γ* in Equation 48) in a data-driven manner (Marshall et al., 2016). In these algorithms, thresholding on neural avalanches before analyzing the shape collapse is a standard preprocessing scheme to control noises (e.g., set an avalanche size threshold and remove all data below the threshold) (Marshall et al., 2016; Papanikolaou et al., 2011). While experimental noises are partly limited, unexpected excursions of scaling parameters away from theoretical predictions may occur after thresholding as well (Laurson, Illa, & Alava, 2009). To our best knowledge, the effects of thresholding on brain criticality analysis are nonnegligible. Although being highly practical, thresholding may lead to significant transient effects to cloud the true scaling property (Villegas, di Santo, Burioni, & Muñoz, 2019). Therefore, any qualitative evaluation of collapse shape after thresholding is questionable regardless of its practicability. Although an ideal approach requires further explorations, we suggest researchers to consider the following methods: (1) estimate *γ* by area fitting (e.g., follow Equation 47 in scaling relation analysis) and collapse shape fitting (e.g., follow Equation 48 in collapse shape analysis), respectively; (2) compare between *γ* derived by these two kinds of fitting and measure the difference. Search for a threshold that minimizes the difference (e.g., makes variation amplitude < 1%) and maintains a reasonable sample size (e.g., maintains > 80% samples); (3) given the chosen threshold and corresponding *γ*, measure the difference (e.g., the dynamic time warping; Keogh & Pazzani, 2001) between 〈*S*(*t*|*T*)〉*T*^1−*γ*^ derived on neural avalanches with different lifetime *T* in the plot of 〈*S*(*t*|*T*)〉*T*^1−*γ*^ vs. $\frac{t}{T}$. Denote the shape collapse error as the averaged difference. Combining these three steps, researchers may partly avoid the errors implied by subjective judgment. Similar ideas can be seen in Marshall et al. (2016).

### Estimating the Slow Decay of Autocorrelation

Finally, the analysis of slow decay of autocorrelation is also error-prone in practice. Although this approach is practical and has been extensively applied (e.g., Pausch et al., 2020; Wilting & Priesemann, 2019), the criterion to determine if the decay is truly slow (e.g., *χ* > 0 in Equation 54 and *ξ* > 0 in Equation 54 are sufficiently small) remains ambiguous. A fixed criterion (e.g., *χ*, *ξ* < 0.5) may serve as an explicit condition of a slow decay. However, this presupposed criterion may deviate from real situations. For instance, the baseline of decay rate in a noncritical brain may be essentially high (e.g., *χ*, *ξ* > 10). Even though the decay rate drops significantly when the brain becomes critical (e.g., *χ*, *ξ* ≃ 1), the presupposed criterion is still unsatisfied and leads to unnecessary controversies on criticality hypothesis. Given that *ξ* is principally independent from spatial subsampling on neurons or brain regions at criticality (Pausch et al., 2020), we suggest researchers to consider the following approaches: (1) do spatial subsampling in both critical and non-ritical brains to derive two groups of *χ* or *ξ* (one group for criticality and another group for noncriticality); (2) use appropriate statistic tests (e.g., choose *t* test; Kanji, 2006; Kolmogorov–Smirnov test; Berger & Zhou, 2014; or Wilcoxon-Mann-Whitney test; Fay & Proschan, 2010; according to sample distribution properties) to verify if two groups of *χ* or *ξ* belong to different distributions. Test if the expectation and variance of *χ* or *ξ* drops significantly from the noncritical group to the critical group according to certain effect sizes.

In summary, statistic techniques bridge between brain criticality theory and empirical data. However, misconception and misuse of statistical analyses of neural avalanche properties still occasionally appear in practice. Although existing techniques remain imperfect in brain criticality analysis, we wish that our discussion may inspire future studies.

## BRAIN CRITICALITY AND OTHER NEUROSCIENCE THEORIES

Ever since brain criticality was introduced into neuroscience, it is frequently speculated as contradictory with other traditional neuroscience hypotheses, such as the conjectured hierarchical processing characteristic of neural information (Felleman & Van Essen, 1991) and the asynchronous-irregular characteristic of neural dynamics (e.g., neurons spike independently in Poisson manners; Burns & Webb, 1976; Softky & Koch, 1993; Stein, Gossen, & Jones, 2005). Meanwhile, the differences between brain criticality and scale-free neural dynamics (Chialvo, 2010; He, 2014; Martinello et al., 2017) are frequently neglected. Before we put an end to our review, we discuss the relations between brain criticality and these neuroscience theories.

### Brain Criticality and Hierarchical Processing

The hierarchical processing of neural information (Felleman & Van Essen, 1991) is initially speculated to contradict critical neural dynamics since hierarchical topology has not been used as an explicit condition to analytically derive criticality (e.g., see derivations in di Santo et al., 2017; García-Pelayo et al., 1993; Gros, 2010; Harris, 1963; Janowsky & Laberge, 1993; Larremore et al., 2012; D. S. Lee et al., 2004; Otter, 1949; Robinson, 2021). On the contrary, random graphs without strict hierarchical structures seem to be more widespread in criticality derivations. Recently, this speculation has been challenged by numerous discoveries of the facilitation effects of hierarchical modular structures on critical dynamics (E. J. Friedman & Landsberg, 2013; Kaiser & Hilgetag, 2010; Rubinov et al., 2011; S. Wang & Zhou, 2012). Meanwhile, computational analysis suggests that information transmission in standard feed-forward networks is maximized by critical neural dynamics (Beggs & Plenz, 2003). Parallel to neuroscience, a recent machine-learning study empirically observes and analytically demonstrates that artificial neural networks, a kind of hierarchical structure, self-organize to criticality during learning (Katsnelson, Vanchurin, & Westerhout, 2021). Therefore, brain criticality is not necessarily contradictory with hierarchical information processing, yet more analyses are required to understand how brain criticality affects hierarchical processing schemes.

### Brain Criticality and Asynchronous-Irregular Characteristic

Brain criticality and the asynchronous-irregular (AI) characteristic may correspond to distinct encoding schemes in the brain (Girardi-Schappo et al., 2021; Wilting & Priesemann, 2019). While AI characteristic can minimize redundancy (Atick, 1992; Barlow et al., 1961; Bell & Sejnowski, 1997; Van Hateren & van der Schaaf, 1998) to improve neural encoding (Van Vreeswijk & Sompolinsky, 1996), brain criticality may optimize encoding performance utilizing a series of reverberations of neural activities (Bertschinger & Natschläger, 2004; Boedecker, Obst, Lizier, Mayer, & Asada, 2012; Del Papa, Priesemann, & Triesch, 2017; Haldeman & Beggs, 2005; Kinouchi & Copelli, 2006; Shew & Plenz, 2013; X. R. Wang, Lizier, & Prokopenko, 2011). The coexistence of empirical evidence of AI and brain criticality characteristics initially confuses researchers since these characteristics are hypothesized as contradictory to each other (Girardi-Schappo et al., 2021; Wilting & Priesemann, 2019). In experiments, AI characteristic is supported by small correlations between the spike rates of different neurons in cortical microcircuits (Cohen & Kohn, 2011; Ecker et al., 2010) and exponential distributions of interspike intervals (Carandini & Stevens, 2004; Kara, Reinagel, & Reid, 2000) while brain criticality characteristic is observed in neural dynamics recorded from multiple species (e.g., awake monkeys; Petermann et al., 2009, anesthetized rats; Gireesh & Plenz, 2008, slices of rat cortices; Beggs & Plenz, 2003; Shew et al., 2009, and humans; Poil et al., 2008). A recent study demonstrates that cortical spikes may propagate at somewhere between perfect criticality (e.g., OC or SOC depending on whether underlying mechanisms are exogenous or endogenous) and full irregularity (Wilting & Priesemann, 2019), similar to the cases of qC and SOqC. Meanwhile, it is known that stimulus drives suppress irregularity in neural activities (Molgedey et al., 1992). These results imply that brain criticality may not necessarily contradict AI characteristic. On the contrary, they may coexist when stimulus drives are too weak to disturb brain criticality (e.g., OC or SOC) and suppress AI characteristics. In our previous discussions, we have analytically proven that neural avalanche exponents, the fundamental properties of brain criticality, can still be derived under the condition of independent neuron activation, a key feature of AI characteristics (Wilting & Priesemann, 2019). This result suggests that brain criticality and AI characteristics do not contradict each other. As for the case where stimulus drives are nonnegligible, a recent study presents an elegant theory to prove that two homeostatic adaptation mechanisms (i.e., the short-term depression of inhibition and the spike-dependent threshold increase) enable synaptic excitation/inhibition balance, AI characteristics, and SOqC to appear simultaneously in the same neural dynamics (Girardi-Schappo et al., 2021). Similarly, it is suggested that neural dynamics with criticality or with AI characteristics can be generated by the same neural populations if the synaptic excitation/inhibition balance is fine tuned appropriately (J. Li & Shew, 2020).

### Brain Criticality and Power Law Behaviors in Neural Dynamics

Neural dynamics with power law behaviors is a necessary but insufficient condition of brain criticality. This property is frequently neglected in practice. Power law behaviors are widespread in nature because they can be generated by diverse mechanisms, such as exponential curve summation and preferential attachment (Mitzenmacher, 2004; Reed & Hughes, 2002). It has been reported that the aggregate behaviors of noncritical stochastic systems may also create scale-free dynamics within a limited range (Touboul & Destexhe, 2010, 2017). In the brain, the generic scale-free properties can be implied by neutral dynamics, a kind of dynamics where the population size of neutral individuals (or dynamically homogeneous individuals) does not tend to increase or decrease after adding a new individual that is neutral to existing ones (see neutral theories for further explanations; Blythe & McKane, 2007; Liggett, 2006). This generic property can generate power law neural avalanches without criticality (Martinello et al., 2017). Meanwhile, bistability phenomena, a kind of fine-tuned or self-organized discontinuous phase transitions with limit cycles rather than critical points, can also create neural dynamics with power law properties (Buendía et al., 2020a; Cocchi et al., 2017; di Santo, Burioni, Vezzani, & Muñoz, 2016). Consequently, we emphasize that neural avalanche exponents alone are insufficient to prove or disprove any brain criticality hypothesis. These power law exponents are meaningless for brain criticality hypothesis unless they satisfy the scaling relation.

## BRAIN CRITICALITY: CONCLUSIONS ON CURRENT PROGRESSES AND LIMITATIONS

Given what have been reviewed above, we arrive at a point to conclude on the current progresses and limitations in establishing theoretical foundations of different types of brain criticality, that is, ordinary criticality (OC), quasi-criticality (qC), self-organized criticality (SOC), and self-organized quasi-criticality (SOqC). As we have suggested, an inescapable cause of various controversies is the nontriviality of physical theories that analytically derive brain criticality and statistical techniques that estimate brain criticality from empirical data. Immoderate omitting of these theoretical foundations, especially their imperfection, in practice may lead to confusions on the precise meaning, identification criteria, and biological corollaries of brain criticality. To address these problems, we have introduced the mainstream theoretical foundations of brain criticality, reformulated them in the terminology of neuroscience, and discussed their error-prone details.

Thanks to the increasing efforts devoted to improving theoretical frameworks of criticality in the brain, researchers have seen substantial progresses in explaining various important neuroscience problems, including but not limited to efficient cortical state transitions (Fontenele et al., 2019), dynamic range maximization in neural responses (Kinouchi & Copelli, 2006; Shew et al., 2009), and optimization of information transmission and representation (Shew et al., 2011). These advances have been comprehensively reviewed by existing works (Beggs, 2007; Chialvo, 2010; Cocchi et al., 2017; Hesse & Gross, 2014; Muñoz, 2018; Shew & Plenz, 2013) and are not discussed in detail in our review. The benefits of studying brain criticality, as we have suggested, lay in the possibility to analyze brain function characteristics with numerous statistical physics theories relevant to brain criticality, such as directed percolation (Hinrichsen, 2000; Lübeck, 2004), conserved directed percolation (Bonachela et al., 2010; Bonachela & Muñoz, 2008), and dynamical percolation theories (Bonachela et al., 2010; Steif, 2009). These theories characterize the brain as a physical system with avalanche behaviors, enabling researchers to analyze various propagation, synchronization, and correlation properties of neural dynamics (e.g., continuous phase transitions). These properties intrinsically shape neural information processing (e.g., encoding; Bertschinger & Natschläger, 2004; Boedecker et al., 2012; Del Papa et al., 2017; Haldeman & Beggs, 2005; Kinouchi & Copelli, 2006; Shew & Plenz, 2013; X. R. Wang et al., 2011; transmission; Shew et al., 2011, and memory; Haldeman & Beggs, 2005; Krotov & Hopfield, 2020) and can be readily recorded in neuroscience experiments. Therefore, the nonequilibrium dynamic processes and potential criticality defined by statistical physics theories are highly applicable to characterizing brain functions. As we have discussed in Figure 2, researchers can consider diverse brain criticality phenomena in neural dynamics by defining different control (e.g., the balance between excitatory and inhibitory neurons; Hardstone et al., 2014; Poil et al., 2012) and order (e.g., active neuron density; Dalla Porta & Copelli, 2019) parameters, corresponding to multifarious biological mechanisms underlying neural dynamics (e.g., synaptic depression; Levina et al., 2007). Meanwhile, the definition of neural avalanches can flexibly change from neural spikes and local field potentials to global cortical oscillations. The flexibility of brain criticality and neural avalanche definitions enables researchers to analyze different functional properties on distinct organizational levels in the brain.

The limited theoretical foundations of brain criticality in the brain, however, have become irreconcilable with their increasingly widespread applications. Although the analytic theories of brain criticality have solid physics backgrounds, they needlessly become black boxes for neuroscientists in practice. On the one hand, the details of brain criticality theory frequently experience immoderate neglecting in neuroscience studies. On the other hand, to our best knowledge, there is no accessible and systematic introduction of the statistical physics foundations of brain criticality in the terminology of neuroscience yet. These obstacles severely impede neuroscientists from comprehensively understanding brain criticality, eventually motivating us to present this review. When we turn to bridging between brain criticality theories and experiments, one can find nonnegligible gaps separating between theories and experiments. Although numerous biological factors (e.g., neural plasticity; De Arcangelis et al., 2006; Levina et al., 2007, 2009; membrane potential leakage; Levina et al., 2007; Millman et al., 2010; Rubinov et al., 2011; Stepp et al., 2015; retro-synaptic signals; Hernandez-Urbina & Herrmann, 2017; spatial heterogeneity; Girardi-Schappo, Bortolotto, Gonsalves, Pinto, & Tragtenberg, 2016; Moretti & Muñoz, 2013; and refractory period; Fosque et al., 2021; Williams-García et al., 2014) have been considered in brain criticality characterization, existing theories more or less suffer from deviations from actual neural system properties. For instance, the requirements of conserved neural dynamics and an infinite timescale separation between the dissipation and drive processes required by SOC may not be biologically realistic (Muñoz, 2018). The implicit requirement of a sufficiently large system size by the mean-field theories of brain criticality may not always be satisfied during neural avalanche recording, implying nonnegligible finite size effects (Girardi-Schappo, 2021). Meanwhile, to precisely verify the existence of a detailed type of brain criticality (e.g., confirm the actual universality class) in empirical neural data is principally infeasible. As we have explained, the common criteria used for brain criticality hypothesis verification, such as neural avalanche exponents (Bauke, 2007; Clauset et al., 2009; Deluca & Corral, 2013; Marshall et al., 2016; Yu et al., 2014; scaling relation; Lübeck, 2004; Lübeck & Heger, 2003; universal collapse shape; Bhattacharjee & Seno, 2001; Laurson et al., 2009; Marshall et al., 2016; Papanikolaou et al., 2011; and slow decay of auto-correlation; Pausch et al., 2020; Wilting & Priesemann, 2019), are derived according to directed percolation theory under mean-field assumptions. Among four types of brain criticality in absorbing phase transitions, only OC originally belongs to directed the percolation universality class, while qC, SOC and SOqC conditionally exhibit directed percolation behaviors. In most cases, one can only verify if the brain is plausibly at criticality (e.g., whether neural avalanches obey universal collapse and have the power law exponents that satisfy the scaling relation). When observed neural avalanche exponents depart from their mean-field approximation results but still satisfy the scaling relation, there may exist an OC phenomenon affected by nonmean-field factors (e.g., network topology; Girardi-Schappo, 2021) or exist a certain qC, SOC, or SOqC phenomenon caused by diverse mechanisms. Additional information of neural dynamics properties is inevitably required to determine the category belonging to the hypothesized brain criticality, which poses daunting challenges to neuroscience experiment designs. Moreover, the potential validity issues of applying the theoretical tools derived from directed percolation theory to verify brain criticality in synchronous phase transitions deserve special attention (for similar opinions see Dalla Porta & Copelli, 2019). It remains controversial if absorbing and synchronous phase transitions robustly share specific features (see reported similarities; Buendía et al., 2021; di Santo et al., 2018; Fontenele et al., 2019; Girardi-Schappo et al., 2021; and differences; Buendía et al., 2021; Fontenele et al., 2019; Girardi-Schappo et al., 2021). Any speculated relations between these two kinds of critical phenomena should be tested with caution. Furthermore, statistic techniques to estimate and verify brain criticality from empirical data are yet imperfect. The estimation of some properties of neural avalanches is error prone in practice and may lead to serious validity issues. Although we suggest compromised solutions to these issues, more optimal approaches are required in future studies.

## BRAIN CRITICALITY: SUGGESTIONS OF FUTURE DIRECTION

We submit that this review not only summarizes the latest developments in the field of studying criticality in the brain, but also serves as a blueprint for further explorations. Below, we offer concrete recommendations of future directions.

First, we suggest researchers carefully rethink the theoretical foundations of criticality in the brain. Immoderately omitting these foundations in neuroscience needlessly muddies an already complex scientific field and leads to potential validity issues. While we have presented a self-contained framework of brain criticality to characterize neural dynamics as a physical system with avalanches, plentiful details are uncovered in this article (e.g., the Landau–Ginzburg theory; di Santo et al., 2018) because the statistical physics theories of brain criticality are essentially grand. We recommend researchers to further improve our work and explore a more accessible and systematic reformulation of related physics theories, such as directed percolation, conserved directed percolation, dynamic percolation, and nonequilibrium dynamics, in the context of neuroscience. Moreover, we note that these theories are not initially proposed for brain analysis. It is normal to see gaps between these theories and real situations of the brain. We urge researchers to develop new variants of criticality formalism that is more applicable to the brain or even explore new universality classes of continuous phase transitions.

Second, neuroscience is in urgent need of new physical theories and statistical techniques to bridge between brain criticality hypotheses and experiments. Although existing theories and techniques have become increasingly widespread and cover most of the pivotal details of brain criticality, there remain various limitations, as we have suggested. Specifically, we suggest five potential directions to resolve these problems: (1) combine brain criticality theories with large-scale neural dynamics recording or computation to include more realistic biological details into brain criticality theories and establish a closer connection with experimental observations; (2) try to summarize, standardize, and subdivide these theories according to the concrete biological meanings of brain criticality phenomena, prerequisites of model definitions, and scopes of application—try to avoid abusing or misusing of different brain criticality theories; (3) develop open-source toolboxes of theoretical models and statistical techniques to routinize brain criticality analysis in neuroscience studies (one can see existing efforts to achieve this objective; Marshall et al., 2016); (4) establish open-source, multispecies, and large-scale datasets of neural dynamics recorded from both critical and noncritical brains—validate different statistic techniques of brain criticality estimation and testing on these datasets and, more importantly, confirm appropriate baselines to define the criteria of brain criticality identification (see notable contributions in Girardi-Schappo, 2021); (5) explore new nonequilibrium statistical physics theories for synchronous phase transitions or analytically verify the theoretical validity of directed percolation formulation of synchronous phase transitions.

Third, parallel to neuroscience, the discoveries of critical phenomena in other learning and computation systems also merit attention. Learning or computing at the edge of chaos has been proven as a mechanism to optimize the performance of learners (e.g., recurrent neural networks; Bertschinger & Natschläger, 2004). The well-known residual connections can control the performance degradation of artificial neural networks because they enable networks to self-organize to criticality between stability and chaos to preserve gradient information flows (Yang & Schoenholz, 2017). It is recently demonstrated that any artificial neural network generally self-organizes to criticality during the learning process (Katsnelson et al., 2021). In the future, it would be interesting to explore whether information processing processes in brains and artificial neural networks can be universally characterized by a unified criticality theory.

Overall, we anticipate the potential of well-validated studies of criticality in the brain to greatly deepen our understanding of neural dynamics characteristics and their roles in neural information processing. Laying solid theoretical foundations of studies is the most effective and indispensable path to contributing to this booming research area.

## ACKNOWLEDGMENTS

Authors are grateful for discussions and assistance of Drs. Yaoyuan Wang and Ziyang Zhang from the Laboratory of Advanced Computing and Storage, Central Research Institute, 2012 Laboratories, Huawei Technologies Co. Ltd., Beijing, 100084, China.

## AUTHOR CONTRIBUTIONS

Yang Tian: Conceptualization; Formal analysis; Investigation; Methodology; Visualization; Writing – original draft; Writing – review & editing. Zeren Tan: Formal analysis; Methodology. Hedong Hou: Formal analysis; Methodology. Guoqi Li: Validation; Writing – review & editing. Aohua Cheng: Formal analysis; Writing – review & editing. Yike Qiu: Validation; Writing – review & editing. Kangyu Weng: Validation; Writing – review & editing. Chun Chen: Validation; Writing – review & editing. Pei Sun: Conceptualization; Project administration; Supervision; Validation; Writing – original draft; Writing – review & editing.

## FUNDING INFORMATION

Pei Sun, The Artificial and General Intelligence Research Program of Guo Qiang Research Institute at Tsinghua University, Award ID: 2020GQG1017.

## TECHNICAL TERMS

Neurophysics: A branch of biophysics that develops and uses physics theories to study the neural system.
Brain connectivity: Refers to the anatomical connectivity formed by synaptic connections and the functional connectivity formed by dynamic interactions among neurons.
Statistical physics: A branch of theoretical physics that develops mathematical theories of the characterization and approximations of large populations with inherently stochastic natures.
Percolation theory: A physics theory that characterizes critical phenomena and phase transitions from a probabilistic and geometric perspective.
Hopfield network: An Ising model of a neural network, which serves as a content-addressable memory system with binary nodes or continuous variables.
Information thermodynamics: A branch of statistical physics that develops mathematical theories of the exchange between information quantities and thermodynamic quantities.
Dynamic range: The intensity or amplitude range of stimulus inputs that is encoded by the given neural dynamics.
Fine tuning: A concept in theoretical physics, which refers to the case where system parameters must be precisely manipulated in order to fit with certain observations.
Discontinuous phase transition: A kind of phase transition where the first-order derivative of the order parameter diverges when transition happens.
